# A low-carbon electricity sector in Europe risks sustaining regional inequalities in benefits and vulnerabilities

**DOI:** 10.1038/s41467-023-37946-3

**Published:** 2023-04-18

**Authors:** Jan-Philipp Sasse, Evelina Trutnevyte

**Affiliations:** Renewable Energy Systems, Institute for Environmental Sciences (ISE), Section of Earth and Environmental Sciences, University of Geneva, Uni Carl Vogt, Boulevard Carl Vogt 66, CH-1211 Geneva 4, Switzerland

**Keywords:** Energy modelling, Renewable energy, Energy justice

## Abstract

Improving equity is an emerging priority in climate and energy strategies, but little is known how these strategies would alter inequalities. Regional inequalities such as price, employment and land use are especially relevant in the electricity sector, which must decarbonize first to allow other sectors to decarbonize. Here, we show that a European low-carbon electricity sector in 2035 can reduce but also sustain associated regional inequalities. Using spatially-explicit modeling for 296 sub-national regions, we demonstrate that emission cuts consistent with net-zero greenhouse gas emissions in 2050 result in continent-wide benefits by 2035 regarding electricity sector investments, employment gains, and decreased greenhouse gas and particulate matter emissions. However, the benefits risk being concentrated in affluent regions of Northern Europe, while regions of Southern and Southeastern Europe risk high vulnerabilities due to high adverse impacts and sensitivities, and low adaptive capacities. Future analysis should investigate policy mechanisms for reducing and compensating inequalities.

## Abstract (web summary)

The low-carbon electricity sector in Europe can bring overall benefits of new investment, employment, and decreased emissions, but could sustain regional inequalities between Northern and Southern Europe.

## Introduction

Equity is increasingly mentioned as one of the priorities in current energy and climate strategies, such as the Biden administration’s $2 trillion infrastructure plan for the US^1^ or the European Green Deal^2^. Although a low-carbon energy transition promises many benefits such as reduced air pollution^3^ and new employment opportunities^4^, historical examples show that energy transitions can exacerbate inequalities. For instance, the decline of the German coal sector since 1960 affected 600,000 jobs, mostly concentrated in the Ruhr region^5^. More recently, the 2020 oil price drop and the COVID-19 pandemic led to regional employment losses in energy sectors other than coal too^6^. In anticipation of rising equity concerns, in 2018 the European Commission has emphasized the importance of not leaving any EU citizens or regions behind^7^ in its net-zero emissions strategy, and further established the Just Transition Mechanism to mobilize at least €100 billion by 2027 for the most affected regions^8^.

While current equity discussions almost exclusively focus on employment losses in mining^9,10^, the adverse and beneficial impacts of the transition are broader. Not only will mines need to close, but so will large fossil fuel-based generation facilities, leading to divestment^11^, employment losses^5,9^, decreased local tax revenue^12,13^, and reduced overall attractiveness of some regions to new investment^14^. Potential increases in energy prices will disproportionally affect regional competitiveness^15^ and low-income households who are least able to adapt to these adverse impacts and are therefore most vulnerable^16–18^. Large-scale renewable generation and associated grid requirements will place uneven spatial pressure on land use and landscape quality^19,20^. While some regions will be able to adapt to adverse impacts, such as higher electricity prices^18^ and employment losses^21^, other regions will benefit from additional investments^22^ and reduced air pollution^23–26^ related to zero-carbon infrastructures. Nevertheless, the regional balances of benefits and vulnerabilities are unknown upfront.

The multifaceted nature of regional benefits and vulnerabilities due to the transition requires a holistic framework that accounts for multiple impacts under the lens of equity. So far, most regionalized analyses have focused on showing that zero-carbon electricity systems are technically and economically viable without anticipating directly associated impacts^27–30^. Other studies focused on quantifying regionalized impacts, such as investment, employment, air pollution, health effects, and land use, without taking sensitivities and adaptive capacities into account^22,23,26,31^. Among more holistic studies, Carley and colleagues^32^ developed a vulnerability index for the case of historic price impacts of Renewable Portfolio Standards across US counties but did not include other vulnerabilities and benefits. There have been more conceptual proposals that analysis of regional inequalities should guide equitable climate and energy policy^33–35^. What is still missing is a framework for regionalized quantification of benefits and vulnerabilities, combined with prospective modeling of infrastructures and equity analysis.

Here, we develop such a framework for assessing regional benefits and vulnerabilities linked to a low-carbon transition of the European electricity sector. This sector is policy relevant as it has high greenhouse gas emissions^36^ and is expected to decarbonize first to allow other sectors to decarbonize before 2050^37,38^. The electricity sector also has the largest share of 63% of all energy sector employment within the EU^39,40^, which can be expected to grow when Europe transitions to a more electrified system. Our novel modeling framework advances the literature on just transitions by adapting the vulnerability framework from climate change adaptation literature^41–43^ and by combining multi-dimensional benefit-vulnerability analysis with prospective sub-national electricity sector modeling. Drawing on social scientific concepts of equity^34,44,45^, we assess benefit and vulnerability related to the transition to a low-carbon electricity sector across 296 NUTS-2 regions^46^ in 33 countries. Using the EXPANSE model^22,47,48^ and Modeling to Generate Alternatives (MGA)^22,49,50^ method, we compute 249 minimum cost and near-minimum cost scenarios of the electricity sector in 2035 that put Europe on track to reach its net-zero emissions goal in 2050, and compare these alternative scenarios with a scenario that represents the current electricity system. With this comparison, we quantify regional benefits and adverse impacts regarding investment and divestment, annual average electricity prices, employment, greenhouse gas and particulate matter emissions, and land use. These impacts are central to existing concepts of equity^34,44,45,47^ and have broad effects on regional economies^51^, household budgets^18^, climate mitigation^52^, human mortality^53^, and land use^54^. We then quantify regional vulnerability by combining modeled adverse impacts with proxy indicators that represent regional sensitivity and adaptive capacity to these adverse impacts (overview of concepts is shown in Table 1 of Methods, software to process and visualize model results is provided on Zenodo^55^). Finally, we quantify composite indices of benefit and vulnerability that combine all the impacts. We find that at the continent level, a low-carbon electricity sector primarily brings benefits regarding electricity sector investment, new employment, and decreased emissions. However, these benefits risk being again concentrated in rather affluent regions of Northern Europe, while regions of Southern and Southeastern Europe risk higher vulnerabilities due to high adverse impacts, higher sensitivities, and lower adaptive capacities to the adverse impacts.

## Results

### Spatially explicit scenarios of electricity system infrastructure and its impacts

**Fig. 1:**
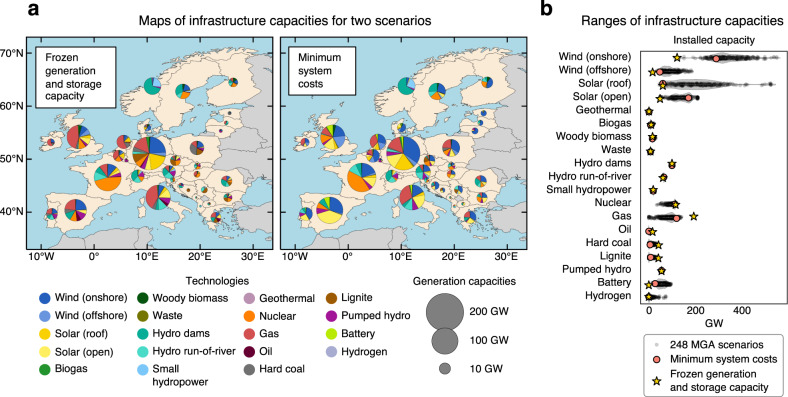
Analyzed scenarios of electricity system infrastructure in 2035. **a** Maps of electricity generation and storage capacities for the scenario of frozen generation and storage capacity and the scenario of minimum system costs. The frozen scenario assumes that generation and storage capacities of 2035 are as in 2018, but that transmission capacities can increase to accommodate the higher electricity demand of 2035. The scenario of minimum system costs is found through optimization. For visualization purposes, installed capacities are shown at country-level instead of NUTS-2 level^46^. Background maps: Made with Natural Earth. Detailed maps of installed capacities at grid node level are shown in Supplementary Fig. 1. **b** Ranges of electricity generation and storage capacities for 248 MGA scenarios in 2035 and the two scenarios: frozen generation and storage capacity, and minimum system costs. Violin plots show the range of capacities for 248 MGA scenarios. MGA scenarios have up to 20% higher total system costs than the minimum cost scenario (see Methods). MGA scenarios are exploratory and do not define national policy targets, subsidies, or taxes. Software to reproduce this figure can be found on Zenodo^55^. MGA Modeling to Generate Alternatives. Source data.

Across all 250 scenarios, the range of infrastructure capacities that drive electricity sector benefits and vulnerabilities vary substantially both spatially and by generation type (Fig. 1). As one example, the frozen scenario, which represents the continuation of the current electricity system, relies on generation capacities that are concentrated in several regions: coal in Germany and Poland, gas in Italy, the Netherlands, Spain, and the United Kingdom, hydropower in Austria, Norway, and Switzerland, and nuclear power in France. In comparison, the low-carbon scenario that minimizes total system costs includes new generation capacities for onshore wind (168 GW), offshore wind (31 GW), and open-field solar PV (122 GW), but not for rooftop solar PV, which remain at comparable levels of 2018. This minimum cost scenario also keeps most nuclear generation capacities of 2018 but reduces those from coal, oil, and gas to achieve emission reduction consistent with the target of European Green Deal in 2050. These capacity reductions mostly occur in Germany, Poland, Spain, and the United Kingdom, where new solar PV and wind capacities are more cost-efficient.

In contrast to the scenario of minimum system costs, the 248 other low-carbon MGA scenarios indicate the feasibility of a more or also less ambitious growth of renewable generation capacities, combined with a more or also less ambitious decline of fossil fuel and nuclear generation capacities. Within MGA constraints, solar PV on rooftops (60–523 GW) and open-field (48–209 GW) as well as onshore (121–549 GW) and offshore (28–185 GW) wind turbines allow for the most ambitious growth. Generation capacities of biomass, geothermal, and hydropower remain at comparable levels of 2018, pointing to limited feasibility of cost-efficiently expanding these technologies. We find that it is feasible to eliminate all fossil fuel generation capacities until 2035, but not nuclear power. Battery power capacities of up to 95 GW (570 GWh energy capacity) and hydrogen power capacities of up to 75 GW (12.6 TWh energy capacity) appear in some scenarios, especially those with high shares of variable renewable generation. Overall, these MGA capacity ranges are in line with capacities of scenarios included in the IPCC’s Sixth Assessment Report (AR6)^56^ that aim for net-zero greenhouse gas emissions in Europe by 2050.

**Fig. 2:**
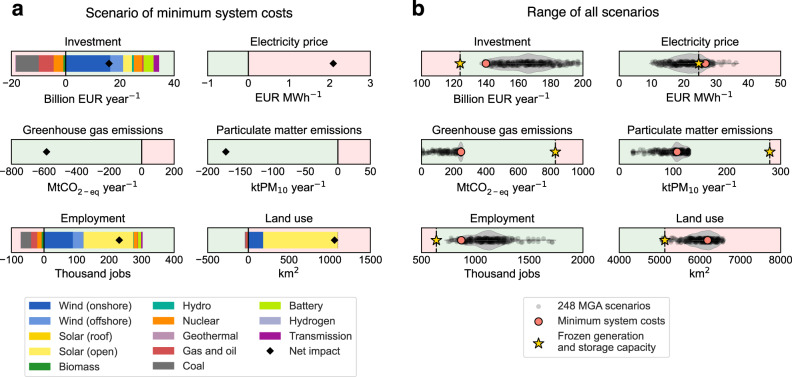
Continent-wide electricity sector impacts for all scenarios in 2035. **a** Benefits and adverse impacts associated with the electricity sector for the scenario of minimum system costs, as compared to the scenario of frozen generation and storage capacity. Impacts on electricity prices are calculated as weighted annual average prices across all 296 NUTS-2 administrative regions^46^ with electricity demand as weights. Impacts on greenhouse gas and particulate matter emissions are calculated as net impacts across all technologies and regions. Impacts on investment, employment, and land use are calculated as continent-wide sums of technology-specific benefits and adverse impacts separately. Benefits are highlighted in green and adverse impacts are highlighted in red. Impacts on investment and annual average electricity prices are endogenously calculated by the EXPANSE model, while impacts on greenhouse gas emissions, particulate matter emissions, employment, and land use are calculated with corresponding factors (see Methods). **b** Range of total impacts associated with the electricity sector for all scenarios, including 248 MGA scenarios and the two scenarios of frozen generation and storage capacity and minimum system costs. Violin plots show the range of total impacts for 248 MGA scenarios. Dashed lines show total impacts of the scenario of frozen generation and storage capacity, which represents a continuation of the current electricity system (see Methods). Software to reproduce this figure can be found on Zenodo^55^. MGA Modeling to Generate Alternatives. Source data.

Such low-carbon electricity sector scenarios lead to associated continent-wide benefits and adverse impacts (Fig. 2). By comparing minimum cost and frozen scenarios (Fig. 2a), the minimum cost scenario suggests additional annualized investment of 34 billion EUR year^−1^, primarily for new generation capacities of onshore and offshore wind power. The minimum cost scenario also includes divestment of 18 billion EUR year^−1^, mostly by closing coal, gas, and nuclear power plants to achieve emission targets. Even with increased electricity generation of solar PV and wind capacities which have low marginal costs, annual average electricity prices across Europe are still 2 EUR MWh^−1^ higher due to high gas generation capacities and exposure to natural gas prices. Net decreases in greenhouse gas emissions of 584 MtCO_2-eq_ year^−1^ and net decreases in particulate matter emissions of 172 ktPM_10_ year^−1^ are mostly linked to decommissioned coal generation capacities. Direct employment gains of the electricity sector of 302 thousand jobs (includes jobs in construction, installation, operation, maintenance, and decommissioning of power plants, but not in manufacturing, fuel extraction, and transport) are mainly associated with open-field solar PV and onshore wind, while direct employment losses of 71 thousand jobs are again mostly associated with coal, gas, and nuclear power plants. Direct land use increases of 1101 km^2^ (excluding land used for fuel extraction and transport) are mainly associated with open-field solar PV and onshore wind, while direct land use decreases of 42 km^2^ are much lower and occur due to reduced use of biomass, coal, gas, oil, and nuclear power plants.

By assessing ranges of total direct impacts of the electricity sector for 248 MGA scenarios and the minimum cost scenario (Fig. 2b), total annualized investments for these scenarios are between 139–197 billion EUR year^−1^ and total direct electricity sector employment is between 0.748–1.714 million jobs. As compared to the frozen scenario, such ranges imply an increase in annualized investment by 15–73 billion EUR year^−1^ and an increase in direct employment by 0.110–1.076 million jobs. Annual average electricity prices of MGA and minimum cost scenarios are 10–36 EUR MWh^−1^, suggesting the possibility of both higher and lower annual average prices as compared to the frozen scenario (24 EUR MWh^−1^). Total greenhouse gas and particulate matter emissions of MGA and minimum cost scenarios are 6–245 MtCO_2-eq_ year^−1^ and 25–129 ktPM_10_ year^−1^ respectively. As compared to the frozen scenario, such ranges imply a decrease by 584–823 MtCO_2-eq_ year^−1^ in greenhouse gas emissions and a decrease by 151–254 ktPM_10_ year^−1^ in particulate matter emissions. Total direct land use of MGA and minimum cost scenarios is 5075–6564 km^2^, indicating mostly increases but also possible small decreases in land use as compared to the frozen scenario (5130 km^2^). These results show that on the continent level, a low-carbon electricity system in Europe in 2035 would primarily bring associated benefits in terms of electricity sector investment, employment gains, and emissions reductions, even if this may not be the case for individual regions. Continent level annual average electricity prices and total land use are less conclusive though, as they depend on each low-carbon scenario.

### Regional electricity sector benefits and vulnerabilities

**Fig. 3:**
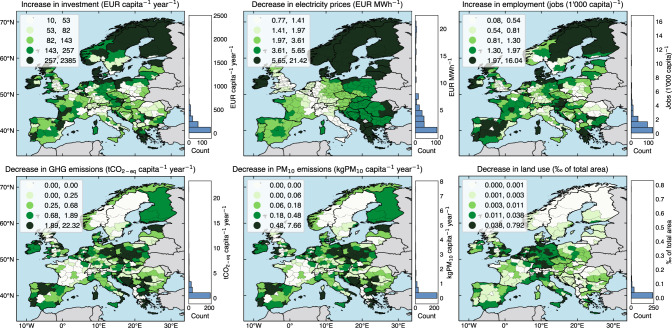
Maps of scenario averages of regional electricity sector benefits in 2035. These maps show the average regional benefits associated with low-carbon electricity sector across 248 MGA scenarios and the minimum cost scenario, which have improved impacts (benefits), as compared to the scenario of frozen generation and storage capacity. The color legend uses a quantile classification scheme so that each category has an equal number of regions. The histograms show the count of regions that lie within 20 equally sized bins. Software to reproduce this figure can be found on Zenodo^55^. GHG greenhouse gas emissions, PM particulate matter, MGA Modeling to Generate Alternatives. Background maps: Made with Natural Earth. Source data.

On a regional level, we find that scenario averages of electricity sector benefits in 2035 (i.e., comparing MGA and minimum cost scenarios against the frozen scenario) vary significantly by region (Fig. 3) and are associated with specific technologies. Highest average benefits regarding additional annualized investment of 257–2385 EUR capita^−1^ year^−1^ are concentrated in regions with high renewable energy resources and available land for new wind capacities, as well as in regions that foresee an expansion of nuclear power. High renewable resources are located near windy coasts, such as in Denmark and Scotland, and in the sunny southern regions, such as in Spain and Greece. Land availability is high in less populated northern regions of Scandinavia. Nuclear capacity expansions are considered in Finland, France, Poland, Slovakia, and the United Kingdom. Highest average benefits due to decreases in annual average electricity prices of 5–21 EUR MWh^−1^ are found outside or on the periphery of Continental Europe, such as the Baltics, Greece, Ireland, and Scandinavia, often due to an increase in electricity generation with low marginal generation costs from wind and solar PV. Highest average benefits regarding direct electricity sector employment gains of 2–16 jobs (1000 capita)^−1^ are in similar regions as the highest investment, but employment gains are associated with solar PV rather than with wind power. Thus, as compared to investment, employment gains occur more in southern parts of Europe with high solar PV potentials in addition to windy coasts with high wind potentials.

Highest average electricity sector benefits regarding net decreases in greenhouse gas emissions of 1.9–22.3 tCO_2-eq_ capita^−1^ year^−1^ are concentrated in regions that currently generate a lot of electricity with coal, gas, and oil power plants in the Balkans, Czech Republic, Germany, Poland, Portugal, Spain, and the United Kingdom. These regions have cost-efficient options to substantially reduce greenhouse gas emissions, which is a benefit as it represents these regions’ ability to cost-efficiently contribute to global climate change mitigation. Highest average benefits regarding net decreases in particulate matter emissions of up to 7.6 kgPM_10_ capita^−1^ year^−1^ are found in similar regions as those with highest average decreases in greenhouse gas emissions due to decreased electricity generation from coal, gas, and oil power plants. Highest average benefits regarding decreased direct land use of up to 0.8‰ of total area are in Belgium, Czech Republic, France, Germany, Italy, Netherlands, Spain, Switzerland, and the United Kingdom due to decommissioned biomass, coal, gas, and nuclear generation capacities.

**Fig. 4:**
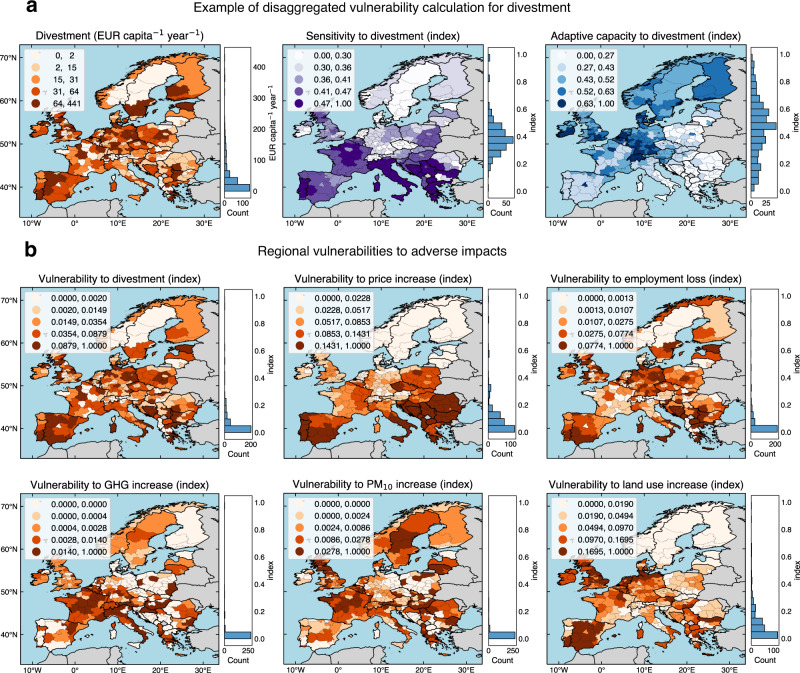
Maps of scenario averages of regional electricity sector vulnerabilities in 2035. **a** An illustrative example of disaggregated vulnerability calculation for divestment associated with the electricity sector. Regional vulnerability combines scenario averages of modeled adverse impact (divestment) with indicators of regional sensitivity and adaptive capacity to divestment. The left map shows the average regional electricity sector divestment across 248 MGA scenarios and the minimum cost scenario as compared to the scenario of frozen generation and storage capacity. **b** Maps of regional vulnerabilities (indices) for six types of adverse impacts associated with the electricity sector: divestment, price increases, employment losses, and increases in greenhouse gas (GHG) emissions, particulate matter (PM_10_) emissions, and land use. Vulnerability indices range from 0 (low) to 1 (high). The color legends in panels (**a**) and (**b**) use a quantile classification scheme so that each category has an equal number of regions. The histograms show the count of regions that lie within 20 equally sized bins. Software to reproduce this figure can be found on Zenodo^55^. MGA Modeling to Generate Alternatives. Background maps: Made with Natural Earth. Source data.

Regional vulnerabilities of low-carbon electricity sector combine high scenario averages of modeled adverse impacts (i.e., comparing MGA and minimum cost scenarios with the frozen scenario) with indicators of high sensitivity and low adaptive capacity to adverse impacts (see Methods). Highest average adverse impacts regarding divestment of 64–441 EUR capita^−1^ year^−1^ (Fig. 4a, Supplementary Fig. 2) are scattered across Europe with some concentration in Germany, Greece, Poland, and Spain, due to phase outs of coal, gas, oil, and nuclear power plants. Regions that we consider most sensitive to divestment (sensitivity index 0.47–1.00, indicators in Table 1, detailed in Methods) are currently economically dependent on the electricity sector, have high government debt, and low GDP, for example, the Balkans, Italy, and Portugal. Regions that we consider most able to adapt to divestment (adaptive capacity index 0.63–1.00) currently have high employment in high-tech sectors, high government quality, and good transport infrastructure, for example, in Belgium, Ireland, South of Germany, Netherlands, Switzerland, and the capital regions of Western Europe. We consider regions with combinations of high divestment, high sensitivity, and low adaptive capacity to divestment as most vulnerable to divestment. These regions (vulnerability index 0.088–1.000) are scattered across Europe but are predominantly located in Spain, and parts of the Balkans, Germany, and Poland (Fig. 4b), due to closures of coal, oil, gas, and nuclear power plants.

We find regional disparities in electricity sector vulnerabilities for all types of adverse impacts (Fig. 4b). Regions that are most vulnerable to increases in annual average electricity prices (vulnerability index 0.143–1.000) are in the Balkans, Eastern Europe, Portugal, and South of Italy and Spain. These vulnerabilities are primarily attributed to increases in electricity prices due to exposure to gas prices as well as high sensitivity (e.g., due to high poverty risk) and low adaptative capacity to these increased electricity prices (e.g., due to low housing benefits). Regions that are most vulnerable to employment losses (vulnerability index 0.077–1.000) are primarily in regions with planned or unplanned closures of existing fossil fuel and nuclear power plants where there are combinations of high employment losses as well as high sensitivity (e.g., due to high long-term unemployment rates) and low adaptive capacity to employment losses (e.g., due to low unemployment benefits). Regions with planned closures of existing nuclear power plants are in Belgium and Germany. Regions with unplanned closures of existing fossil fuel and nuclear power plants are in, for example, Bosnia Herzegovina and the United Kingdom.

The most vulnerable regions in terms of net increases in greenhouse gas emissions and hence climate mitigation (vulnerability index 0.014–1.000) are predominantly located in Italy and France due to increased electricity generation from gas (representing difficulties to further reduce emissions) as well as high sensitivity (e.g., due to high climate-related fatalities) and low adaptive capacity (e.g., due to high shares of vulnerable population groups). The most vulnerable regions to increased particulate matter emissions (vulnerability index 0.028–1.000) are scattered across Europe, with some concentration in Eastern Europe, Sweden, France, and the United Kingdom. These regions have combinations of increased particulate matter emissions attributed to local increases in electricity generation from biomass as well as high sensitivity (e.g., due to high years of life lost from particulate matter emissions) and low adaptive capacity to these increased emissions (e.g., due to low healthcare benefits). The most vulnerable regions to increases in direct land use (vulnerability index 0.169–1.000) are mostly in Spain and coastal regions of the Netherlands, Germany, and the United Kingdom. These regions are most vulnerable due to high land use associated with new open-field solar PV and onshore wind capacities across modeled scenarios as well as high sensitivity (e.g., due to high population density) and low adaptive capacity to land use (e.g., due to low land availability).

### Composite electricity sector benefit and vulnerability indices and scenario variability

**Fig. 5:**
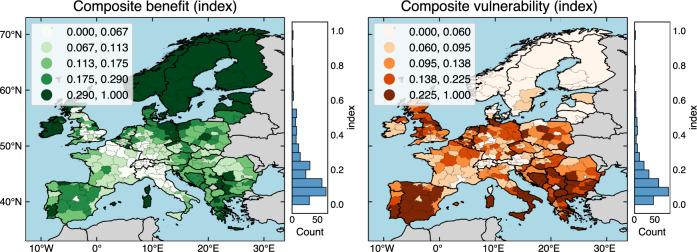
Maps of composite indices of electricity sector benefit and vulnerability. Composite indices combine findings on all six impact types across 248 MGA scenarios and the minimum cost scenario as compared to the frozen scenario of 2035 (details are provided in Methods). Composite benefit and vulnerability indices range from 0 (low) to 1 (high). These two composite indices are treated separately because various benefits and vulnerabilities do not necessarily compensate each other, for example, due to differences in required skill sets of employment associated with low-carbon and fossil-fuel infrastructure. Therefore, regions can have both high benefits and high vulnerabilities. The color legends use a quantile classification scheme so that each category has an equal number of regions. The histograms show the count of regions that lie within 20 equally sized bins. Software to reproduce this figure can be found on Zenodo^55^. MGA Modeling to Generate Alternatives. Background maps: Made with Natural Earth. Source data.

By creating composite electricity sector benefit and vulnerability indices across all impact types (Fig. 5; see Methods), we find that benefits of low-carbon scenarios occur mostly in regions of Northern Europe, while vulnerabilities occur mostly in regions of Southern and Southeastern Europe. Regions with highest composite benefit indices are primarily located in the Baltics, Germany, Ireland, Scandinavia, and Scotland due to new investment and employment gains, and decreased annual average electricity prices, greenhouse gas and particulate matter emissions, and land use. Regions with highest composite vulnerability indices are mostly located in the Balkans, Southern Italy, Portugal, Poland, and Spain due to electricity sector vulnerability to combinations of divestment and employment losses, and increased annual average electricity prices, greenhouse gas and particulate matter emissions, and land use. Regions with a very low composite benefit index, suggesting a lack of benefits, includes some lower income regions of Southern Italy. Regions with very low composite vulnerability index are located mostly in richer regions of Scandinavia and Switzerland. Hence, such a regional distribution of benefits and vulnerabilities across our low-carbon scenarios in 2035 indicates of important risks of sustaining existing regional inequalities across Europe. We confirm the robustness of this finding by applying the Gini index^57^ to spatial distributions of composite benefit and vulnerability of all individual scenarios with varying levels of total greenhouse gas emissions (Supplementary Fig. 3). Scenarios with lowest total greenhouse gas emissions tend to exclude scenarios of highest regional inequality of both composite benefit and vulnerability, meaning that climate mitigation can reduce regional inequalities. However, there is still high variability of inequality outcomes depending on which low-carbon electricity system is implemented and a risk of sustaining existing regional inequalities, especially between Northern and Southern Europe.

**Fig. 6:**
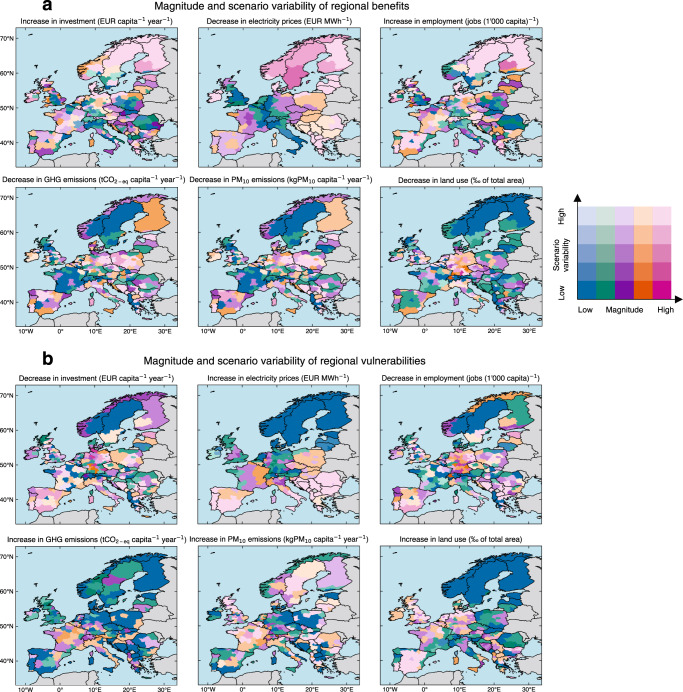
Maps of magnitude and scenario variability of electricity sector benefits and vulnerabilities across all scenarios. Magnitude is defined as the average regional benefit (**a**) or average regional vulnerability (**b**) across all 248 MGA scenarios and the minimum cost scenario as compared to the scenario of frozen generation and storage capacities. Scenario variability is defined as the standard deviation of regional benefit and vulnerability across all scenarios. The color legend in (**a**) and (**b**) uses a quantile classification scheme so that each category has an equal number of regions. Dark red colors indicate high magnitude and low scenario variability of benefit and vulnerability. Light red colors indicate high magnitude and high scenario variability of benefit and vulnerability. Dark blue colors indicate low magnitude and low scenario variability of benefit and vulnerability. Light blue colors indicate low magnitude and high scenario variability of benefit and vulnerability. Software to reproduce this figure can be found on Zenodo^55^. GHG greenhouse gas emissions, PM particulate matter, MGA Modeling to Generate Alternatives. Background maps: Made with Natural Earth. Source data.

To understand the influence of variability across all scenarios on our results, we additionally categorize regions by magnitude and scenario-related spread of benefits and vulnerabilities (Fig. 6). Very few regions have consistently high benefits across all scenarios (Fig. 6a). Regarding electricity sector investment and employment gains, such regions include Estonia due to consistent increases in wind capacities across all scenarios. Regarding decreased annual average electricity prices, such regions include Southern Sweden, due to consistent increases in electricity generation from wind capacities with low marginal costs. Regarding reduced greenhouse gas and particulate matter emissions, such regions include Finland due to consistent decreases in electricity generation from coal. Far more regions with high magnitudes of benefits also have high scenario variability, indicating high uncertainty related to where and which electricity sector infrastructures will be built or closed. Regarding investment, reduced annual average electricity prices, and employment gains, such regions include Scandinavia and Scotland, due to high scenario variability of adding high wind capacities in these regions. Regarding reduced greenhouse gas and particulate matter emissions, such regions include Bosnia and Herzegovina, Germany, and Poland, due to high scenario variability of decommissioning high coal generation capacities. Regarding reduced land use, such regions include Germany, due to high scenario variability of decommissioning high biomass capacities. We also find regions with consistently low benefits across all low-carbon electricity sector scenarios. Regarding electricity sector investment and employment gains, such regions include Southern Poland, due to consistently low wind and solar PV capacities. Regarding reduced annual average electricity prices, such regions include Italy and Switzerland, due to limited potentials to further reduce annual average electricity prices. Regarding decreased greenhouse gas emissions, particulate matter emissions, and land use, such regions include Norway and Sweden, due to limited potentials to further reduce emissions and land use.

Very few regions have consistently high electricity sector vulnerabilities across all scenarios (Fig. 6). Regarding electricity sector divestment and employment losses, such regions include Belgium and Germany, where there are planned closures of nuclear power plants across all scenarios. Regarding increased particulate matter emissions, such regions include Western Austria, due to consistent increases in electricity generation from biomass across all scenarios. Far more regions with high magnitudes of vulnerabilities also have high scenario variability, which indicates the uncertainty of vulnerabilities, depending on the location of new and closed infrastructures. Regarding electricity sector divestment and employment losses, such regions include the Balkans, Germany, and Poland due to high scenario variability of decommissioning coal generation capacities, as well as Spain and South of Italy due to high scenario variability of decommissioning nuclear and gas generation capacities. Regarding increased annual average electricity prices, such regions include the Balkans, Portugal, Southern Italy, and Spain, due to high scenario variability of exposure to high gas prices. Regarding increased greenhouse gas emissions, such regions include Italy, due to high scenario variability of increased electricity generation from gas. Regarding increased particulate matter emissions, such regions include France, due to high scenario variability of increased electricity generation from biomass. Regarding increased land use, such regions include the Netherlands and Spain, due to high scenario variability of increased onshore wind and open-field solar PV capacities. Regions with consistently low vulnerabilities across all types of impacts are predominantly located in relatively affluent regions, such as in Scandinavia and Switzerland, implying both low scenario variability and low magnitude of regional vulnerability.

## Discussion

In our spatial analysis of the European electricity system and associated regional benefits and vulnerabilities, we found that transition to net-zero emissions in 2050 can reduce, but consistently poses risks of sustaining existing regional inequalities associated with electricity sector at least in 2035. Regional benefits, which include electricity sector investment and employment gains amongst others, are concentrated in rather affluent regions of Northern Europe. Regional vulnerabilities, which include increases in annual average electricity prices and electricity sector employment losses amongst others, are concentrated in less economically advantaged regions of Southern and Southeastern Europe. These vulnerabilities are exacerbated by the fact that populations in these regions already have difficulties to pay for electricity and are economically dependent on the electricity sector. Regional differences in benefits and vulnerabilities are overlooked on the continent level, as a low-carbon electricity system in Europe by 2035 would primarily bring benefits regarding electricity sector investment, employment gains, and emissions reductions.

This risk of sustaining existing regional inequalities associated with the electricity sector requires that the discussions on equitable energy and climate policy broaden beyond employment in coal mining regions. As much as 60–85% of employment opportunities in the electricity sector stem from direct jobs (see Supplementary Table 1), making it essential to do analysis beyond mining. Multi-faceted regional benefits naturally act as an encouragement for regions to implement new electricity system infrastructures that are needed to achieve European energy and climate targets. As the benefiting regions can end up again being primarily located in affluent parts of Europe which have more capacity to mobilize the required investment and effort, this increases the feasibility of the transition. The other, currently lower income regions may not only struggle to initiate and sustain the transition, but there is also a risk that they will face increased vulnerabilities of the electricity sector and hence could resist the transition. Given the multi-dimensional nature of benefit and vulnerability, a holistic policy response is needed to dampen adverse impacts and to strengthen sensitivities and adaptive capacities for increasing the regional equality of low-carbon electricity sector transition.

Our results show that some technologies dampen adverse impacts associated with a low-carbon electricity sector. New solar PV and wind capacities in sites with decommissioned nuclear and coal power plants could partly compensate for electricity sector divestment and employment losses. Such sites with legacy infrastructure also benefit from existing transmission lines, available land, and fewer landowners compared to greenfield sites^9^. Regions where these benefits and adverse impacts align geographically include southern Finland and Estonia, while regions with less alignment include Eastern Germany and Poland, indicating a more difficult transition. Replacing gas power plants with new solar PV and wind capacities decreases Europe’s exposure to high electricity prices. A decreased reliance on gas power plants also ameliorates energy security concerns related to Europe’s over-dependence on Russian gas^58^. Increasing renewable electricity generation with new solar PV and wind capacities rather than biomass capacities reduces particulate matter emissions and associated adverse impacts on human health. Encouraging offshore wind and rooftop solar PV rather than onshore wind and open-field solar PV significantly reduces land use at potentially still acceptable costs.

Multiple policy measures could strengthen the sensitivities and adaptive capacities of European regions to become less vulnerable to adverse impacts of a low-carbon electricity sector and hence decrease associated regional inequalities. Our analysis provides the starting point to identify the key regions to focus on: the Balkans, Southern Italy, Portugal, Poland, and Spain. Regarding electricity sector divestment and employment losses, policies include job retraining schemes, enabling early retirement, and attracting new industries^59^. Some jobs from new wind turbines and solar PV panels require similar skill sets as jobs from legacy nuclear and coal power plants^9^, which improves the success of job retraining schemes. The German Ruhr region provides an example of economic restructuring away from coal, but its sustained high unemployment compared to the rest of Germany reflects the difficulty of such a transition^59^. Regarding electricity prices, policies include financial transfers to low-income households, disconnection safeguards, and energy efficiency measures, which are still not widely available across Europe, short-term, and do not address underlying causes of vulnerability^18^. Regarding greenhouse gas and particulate matter emissions, policies include heatwave action plans, improved housing, greening of neighborhoods, promoting walking and cycling, and supporting the elderly^60^. Regarding land use, policies include encouraging community involvement when planning new low-carbon infrastructures^61,62^ and encouraging these infrastructures in regions that are less scenic^63^, less populated^64,65^, and have previous experiences with low-carbon infrastructures^66–68^. The persistence of regional inequalities across our modeled scenarios also highlights the importance of compensation schemes between unaffected and affected regions. One example of such a scheme is the Just Transition Mechanism of the European Union^8^, which focuses on coal mining regions and should be broadened to include all European regions that are vulnerable to electricity sector transition.

Following increased policy interest, research on representing inequalities in energy systems modeling has become a new frontier^69^. This study provides a framework to evaluate multidimensional benefits and vulnerabilities at the local level for many future scenarios of the electricity sector. Future research could extend our methods in seven aspects. First, our analysis focuses on the electricity sector and therefore does not capture benefits and vulnerabilities of low-carbon transition in other sectors. The scope of our framework could be horizontally extended across the other energy sectors, such as heating and transport, and vertically extended across supply chains, such as coal mining and manufacturing of wind turbines. Second, the representation of spillover effects across regions could be improved, such as new jobs created in a region due to new investments in neighboring regions. Third, our visual analysis of regional benefit and vulnerability could be complemented with other types of analysis, such as quantitative comparisons with climate-related vulnerability^70^ and energy poverty indices^71^. Fourth, our MGA algorithm to sample alternative scenarios could be adapted to include more extreme scenarios. Fifth, future research could apply participatory methods, such as stakeholder interviews, to elicit preferred indicators and weights of sensitivity, adaptive capacity, and the impacts that we covered. Sixth, future work could consider uncertainties and long-term change in applied indicators and impact factors, such as regarding employment^4,72–74^. Finally, this analysis could be extended to assess various policy options to maximize the overall benefits of the European low-carbon electricity sector while minimizing inequalities.

## Methods

Our workflow links the EXPANSE electricity sector model^22,47,48^ with a benefit-vulnerability framework for the analysis of regional benefits, adverse impacts, sensitivities, adaptive capacities, and vulnerabilities^41–43^, drawn from existing concepts of equity in literature^34,44,45^ (see the overview in Table 1). Software to process modeling results and create associated figures is available on Zenodo^55^.

**Table 1:** Overview of regional benefits, adverse impacts, sensitivities, adaptive capacities, and vulnerabilities considered in this study

Benefits, adverse impacts, and vulnerabilities	Units	Sensitivity concept	Sensitivity indicator	Units	Adaptive capacity concept	Adaptive capacity indicator	Units
Investment and divestment	EUR year^−1^	Economic dependence on electricity sector^88^	Share of total labor costs associated with electricity sector	%	Ability to attract new investments, especially in high tech sectors^88^	Share of employment in high-tech sectors	%
Investment and divestment	EUR year^−1^	Sensitivity of national government to divestment^93^	Government gross debt as a share of GDP	%	Ability to attract new investments due to logistics efficiency^94^	Motorway density in km per 1000 km^2^ of total area	km km^−2^
Investment and divestment	EUR year^−1^	General economic strength of the region^95^	GDP per capita	PPS capita^−1^	Impartiality and quality of public services^88^	Government quality index	Index
Increases and decreases in electricity prices	EUR MWh^−1^	Difficulty to pay electricity bills^88^	Share of population with arrears on utility bills	%	Government support to cope with high electricity prices^88^	Housing benefits per capita	PPS capita^−1^
Increases and decreases in electricity prices	EUR MWh^−1^	Sensitivity of households to high electricity prices^88^	Share of household expenditure for electricity	%	Ability of households to cope with high electricity prices^88^	Net disposable income per capita	PPS capita^−1^
Increases and decreases in electricity prices	EUR MWh^−1^	General sensitivity to high electricity prices^88^	Share of population at risk of poverty or social exclusion	%	Energy-efficient housing to mitigate sensitivity to high electricity prices^88^	Share of near-zero energy residential buildings	%
Employment gains and losses	Jobs MW^−1^	General sensitivity to high unemployment^96^	Long-term unemployment rate	%	Government support to cope with unemployment^96^	Unemployment benefits per capita	PPS capita^−1^
Employment gains and losses	Jobs MW^−1^	Electricity sector-specific sensitivity to employment losses^88^	Jobs per capita in electricity supply sector	Jobs capita^−1^	Government support to find new employment^88^	Expenditure per capita on labor market policy (e.g., job training)	PPS capita^−1^
Employment gains and losses	Jobs MW^−1^	General sensitivity to loss of full-time jobs^96^	Share of population living in households with very low work intensity	%	Workforce engagement^96^	Economic activity rate (share of economically active population)	%
Increases and decreases in greenhouse gas emissions	MtCO_2-eq_ year^−1^	Heat burden^60^	Average cooling degree days (CDD) per year	CDD	Ability of population to cope with extreme heat^60^	Share of non-vulnerable population (5–75 years old)	%
Increases and decreases in greenhouse gas emissions	MtCO_2-eq_ year^−1^	Fatalities associated with climate change^60^	Cumulative fatalities per capita due to extremely high temperatures	Fatalities capita^−1^	Ability of housing to cope with extreme heat^60^	Share of dwellings equipped with air conditioning	%
Increases and decreases in greenhouse gas emissions	MtCO_2-eq_ year^−1^	Economic losses associated with climate change^60^	Cumulative economic losses per capita due to extreme weather events	PPS capita^−1^	Ability to cope with economic losses of climate change^60^	Insured share of cumulative economic losses due to extreme weather events	%
Increases and decreases in particulate matter emissions	kgPM_10_ MWh_el_^−1^	Health care quality and access^60^	Share of population with self-reported unmet needs for medical and dental care	%	Ability of health system to cope with health impacts^60^	Sickness and healthcare benefits per capita	PPS capita^−1^
Increases and decreases in particulate matter emissions	kgPM_10_ MWh_el_^−1^	Health sensitivity of population^60^	Share of population with self-perceived bad health	%	Capacity of health system^60^	Available hospital beds per capita	Beds capita^−1^
Increases and decreases in particulate matter emissions	kgPM_10_ MWh_el_^−1^	Burden levels of particulate matter emissions^60^	Years of life lost (YLL) per capita attributable to particulate matter emissions	YLL capita^−1^	General health of population^60^	Life expectancy	Years
Increases and decreases in land use	m^2^ MW^−1^	Sensitivity of population to high land use^64,65^	Population density	Person km^−2^	Ability of general population to cope with less available land^64,65^	Share of land not covered by artificial land	%
Increases and decreases in land use	m^2^ MW^−1^	Sensitivity of tourism sector to high land use^63^	Number of tourist arrivals per inhabitant	Arrival capita^−1^	Ability of tourism sector to cope with less available land^63^	Share of population not employed in tourism	%
Increases and decreases in land use	m^2^ MW^−1^	Sensitivity of agricultural sector to high land use^97^	Utilized agricultural area as a share of total area	%	Ability of agricultural sector to cope with less available land^97^	Share of gross value added by non-agricultural sectors	%

Regional benefits and adverse impacts are direct results from the EXPANSE model. We derive proxy indicators of regional sensitivity and adaptive capacity (or ability to adapt) to adverse impacts based on regional statistics data (detailed in Supplementary Methods). Vulnerability is computed by combining modeled adverse impacts with proxy indicators of sensitivity and adaptive capacity. Greenhouse gas emissions reflect exposure to climate change mitigation.

*PPS* purchasing power standard.

### EXPANSE electricity system model

EXPANSE^22,47,48^ is a spatially explicit, bottom-up, technology-rich, single-year optimization model of the European electricity system in 2035 that covers 33 countries (European Union minus Cyprus and Malta and plus Albania, Bosnia and Herzegovina, Montenegro, North Macedonia, Norway, Serbia, Switzerland, and the United Kingdom). The model accounts for operation and capacity planning of electricity generation, storage, and transmission. In this analysis, EXPANSE is configured to six-hour timesteps to ensure the computational tractability of modeling many spatially explicit scenarios. Our sensitivity analysis and a previous modeling study^75^ showed that six-hour timesteps are sufficient to account for temporal variability of renewable electricity generation and storage requirements. EXPANSE has also been validated by participating in a model inter-comparison with two other spatial models and through retrospective modeling^76^. EXPANSE represents electricity generation at the level of 296 NUTS-2 regions^46^ and electricity demand, storage, and transmission at the level of 128 transmission grid nodes^77^. EXPANSE ensures that electricity generation, storage, and transmission balance inelastic electricity demand at each transmission grid node and time step (detailed in Supplementary Methods). We set up the EXPANSE model to include a maximum limit in greenhouse gas emissions of 245 MtCO_2-eq_ year^−1^, which is consistent with 70%^38^ greenhouse gas emissions reduction in 2035 as compared to 2019 and hence is in line with the net-zero emissions target in 2050 of the European Green Deal^2^ (detailed in Supplementary Methods). We do not include other specific national policy targets, subsidies, or taxes (e.g., feed-in tariffs, carbon tax), following the exploratory rather normative application of scenario methodology^78^. Further, we assume that no new nuclear and fossil fuel generation capacities can be built as compared to today, except for currently planned expansions^79^. We also assume that all nuclear generation capacities are decommissioned in Belgium and Germany by 2035. We provide further detailed descriptions of the mathematical formulation of EXPANSE, its software implementation, and of the data sources and assumptions on electricity demand, electricity system infrastructure, resource potentials, and technology characteristics (Supplementary Tables 2–5) in the Supplementary Methods.

The key feature of EXPANSE is that it applies Modeling to Generate Alternatives (MGA)^22,47–49^ to compute a comprehensive set of alternative scenarios within the range of acceptable, near-optimal costs. The principle of MGA is to replace the cost minimization function with a cost constraint so that total system costs would be below a pre-defined value called slack, and then to randomly generate many alternative scenarios within the optimization constraints. Even if MGA is typically framed as an analysis of near cost-optimal scenarios, it is also an efficient tool to cover broad ranges of parametric uncertainty in technology costs, employment factors, emission factors, and other indicators^49,80^. We adapt and set up the MGA algorithm to represent a narrower range of MGA scenarios that simultaneously explore near-optimal spaces in continent-wide impacts on total system costs, employment, greenhouse gas emissions, particulate matter emissions, and land use.

As a result, EXPANSE computes 248 alternative MGA scenarios where the slack is randomly varied from 0 to 20% above cost-optimal total system costs. We choose this number of MGA scenarios to follow other MGA studies with similar spatial and temporal detail^22,81,82^. Computing even more MGA scenarios is bounded by the computational tractability of spatially and temporally detailed, continent-scale electricity system models, such as EXPANSE. Total system costs are defined as the sum of annualized capital and variable costs for generation, storage, and transmission. We select a maximum slack of 20% based on the cost deviations found in a retrospective modeling study^80^ and from other forward-looking MGA models^22,49,50^. To ensure the spread and diversity of MGA scenarios not only in terms of total costs, but also other impacts analyzed in this study, we adapt previous algorithms^83,84^ to apply computer-generated random objective functions for investment variables (i.e., country and technology-specific generation capacities) and computer-generated random constraints on five continent-wide impact objectives (i.e., minimizing total system costs, greenhouse gas emissions, particulate matter emissions, and land use, and maximizing total employment). For example, the objective of one MGA scenario in terms of investment could be to maximize total offshore wind capacity in Denmark and to minimize total nuclear capacity in Switzerland, while meeting computer-generated random constraints for all five impact objectives. Thus, we extend the MGA approach for a single cost objective (i.e., replacing the cost objective with a cost constraint) to multiple impact objectives (i.e., replacing five impact objectives with five impact constraints).

Constraints for all five continent-wide impacts are computer-generated in three steps. The aim of these steps is to ensure the feasibility of all five impact constraints. For example, a cost slack of 1% may enable a reduction of greenhouse gas emissions by 0–30% compared to the minimum cost scenario, but not a total elimination of emissions as in the case of a cost slack of 20%. Thus, each cost slack has an implicit feasible range for constraints on total greenhouse gas emissions, particulate matter emissions, land use, and employment. In the first step, the cost-optimal objective value is found by minimizing total system costs. In the second step, the Pareto frontiers are calculated between the cost minimization and each of the other four objective functions for 12 pre-defined cost slacks (0.5, 1, 2, 3, 4, 5, 7.5, 10, 12.5, 15, 17.5, and 20.0%). These scenarios result in 48 MGA scenarios. The Pareto frontiers represent the implicit feasible range for constraints on emissions, land use, and employment that is associated to each cost slack. In the third and final step, 200 MGA scenarios are computer-generated by randomly selecting a cost slack (e.g., 4.2%) and applying randomly selected constraints within the corresponding implicitly feasible range of the other four impacts. Cost-specific feasible ranges of the four other impacts are interpolated based on the Pareto results of the second step.

In addition to these 248 MGA scenarios and the minimum cost scenario, EXPANSE also computes one so-called frozen scenario. The frozen scenario assumes that generation and storage capacities of 2035 are as in 2018, but that transmission capacities can increase to accommodate the higher electricity demand of 2035. This frozen scenario serves as a baseline scenario to compare regional impacts of 248 MGA scenarios and the minimum cost scenario to impacts of the current electricity system in a harmonized way, with the same costs and other assumptions of 2035. In total, this setup leads to 250 scenarios that we analyze in this study.

### Evaluating regional benefits and adverse impacts

EXPANSE estimates regional impacts that are directly associated with installed capacities and operation of electricity system infrastructure. Regional annualized investment (in EUR year^−1^) is calculated endogenously in EXPANSE and refers to annualized capital investment as well as fixed annual operation and maintenance costs of electricity generation, storage, and transmission. Regional electricity prices (in EUR MWh^−1^) are also calculated endogenously in EXPANSE and refer to annual averages of locational marginal prices^85,86^. Regional employment (in the number of jobs) is calculated by multiplying installed capacities (in MW) of generation, storage, and transmission with factors from previous peer-reviewed studies (in jobs MW^−1^, Supplementary Table 1). Jobs refer to direct jobs in construction, installation, operation, maintenance, and decommissioning, but does not include jobs in manufacturing, fuel extraction (e.g., coal mining), and transport. Shares of included jobs per technology vary between 13 and 97%, where lowest shares are associated with lignite (13%) and hard coal (15%) due to exclusion of mining and highest shares are associated with nuclear power (97%) and hydropower storage (95%). For low-carbon technologies, included jobs cover more than half of the jobs. We do not estimate annual changes in employment factors, but these are implicitly accounted for by the MGA approach as a parametric uncertainty tool^49,80^. Regional greenhouse gas emissions (in MtCO_2-eq_ year^−1^) and particulate matter emissions (in tPM_10_ year^−1^) refer to annual direct emissions from fuel combustion for electricity generation. These emissions are calculated by multiplying annual sums of electricity generation (in MWh year^−1^) with corresponding factors (in tCO_2-eq_ MWh_el_^−1^ and kgPM_10_ MWh_el_^−1^, Supplementary Table 1). Regional land use (in km^2^) refers to direct land used by industrial areas and artificial lake areas occupied by electricity generation and storage infrastructure. We exclude land used for fuel extraction and transport. Regional land use is calculated by multiplying regional installed capacities (MW) with corresponding land use factors (in m^2^ MW^−1^, Supplementary Table 1). These factors of aggregated land use do not differentiate by land use type due to lack of data.

Further, we distinguish between benefits (e.g., employment gains) and adverse impacts (e.g., employment losses) by comparing impacts (e.g., jobs) of each MGA scenario and the minimum cost scenario with the impacts of the frozen scenario that represents a continuation of the current electricity system. For investment, employment, and land use, we calculate regional benefits and adverse impacts separately for each technology. For example, we calculate technology-specific employment gains and losses across scenarios for calculating average regional benefits and adverse impacts. The reasoning of this separate calculation is that, for example, employment gains of solar PV do not directly compensate for employment losses of coal, due to differences in required skill sets^9^. We apply the same reasoning for investment and land use, due differences between technologies in terms of economic, environmental, and landscape impacts. For electricity prices, greenhouse gas emissions, and particulate matter emissions, we consider the net regional impacts across all technologies without separating per technology, because we treat these impacts the same for all technologies.

We define regional decreases in investment and employment as adverse impacts, due to potential negative effects on regional economies^51^. We define regional increases in annual average electricity prices as adverse impacts, due to potential negative effects on households budgets^18^. We define regional increases in land use as adverse impacts, due to potential negative landscape impacts^54^, land use conflicts^87,88^ and biodiversity losses^89^. We define regional decreases in greenhouse gas emissions as benefits, as this represents a region’s ability to cost-efficiently mitigate climate change^52^. We define regional increases in particulate matter emissions as adverse impacts, due to potential negative effects on human mortality^53^.

### Evaluating regional sensitivity and adaptive capacity

We quantify overall measures of sensitivity and adaptive capacity for each region and for each type of adverse impact (e.g., employment losses). Sensitivity measures indicate the overall susceptibility of a region to a specific adverse impact. Adaptive capacity measures indicate the overall ability of a region to cope or mitigate that specific adverse impact. Overall sensitivity and adaptive capacity measures are not direct results of the EXPANSE model but are quantified with indicators from regional statistics data (overview of indicators in Table 1; detailed descriptions of indicators, values, and data sources in Supplementary Tables 6, 7). We calculate overall measures of impact-specific sensitivity and adaptive capacity in three steps. In the first step, we normalize all indicators from newest regional statistics data to values between 0 (low) and 1 (high) by applying min-max normalization^90^. In this way, we make indicators with different units and values comparable with each other. This approach follows the logic of multi-criteria analysis^91^ where relative values of sensitivity and adaptive capacity are more important than absolute values. We assume that these relative indicators do not change until 2035, as we observed only marginal annual changes of indicators in the regional statistics data. For example, between 2009 and 2018, the NUTS-2 regions with highest and lowest GDP per capita were consistently the Western Inner City of London and Northern Albania, respectively. In the second step, using weighted-sum method of multi-criteria analysis^91^, we sum three related normalized indicators (e.g., three indicators that measure sensitivity to employment losses) with equal weights of 1/3. Applying equal weights is the most popular weighting method in multi-criteria decision analysis^91^ and is preferable to subjective weights if there is minimal knowledge of decision-maker preferences^92^. However, future research could provide empirical evidence on the appropriate weighting of these indicators. In the third and final step, we again normalize the obtained result to values between 0 (low) and 1 (high) with min-max normalization^90^. Maps of overall regional sensitivity and adaptive capacity are shown in Supplementary Figs. 4 and 5.

### Evaluating average regional benefit and vulnerability

**Fig. 7:**
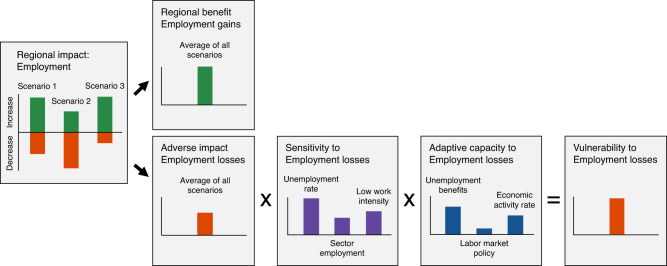
Concept overview of the benefit-vulnerability calculation. Regional impacts are distinguished between benefits and adverse impacts by comparing impacts of each scenario with impacts of the frozen scenario that represents the current electricity system. Regional benefit of each region is computed with Eq. (1) as the average benefit across all scenarios. Regional vulnerability of each region is computed with Eq. (2) that combines average adverse impact across all scenarios with three impact-specific and equally weighted indicators of sensitivity and adaptive capacity.

We calculate scenario averages of regional benefit and vulnerability for each impact type by comparing average benefits and vulnerabilities of 248 MGA scenarios and the minimum cost scenario with the frozen scenario. We calculate benefits and vulnerabilities differently, due to different definitions. We define benefit as being positively affected, which we quantify directly by calculating the modeled scenario average of regional benefit. We define vulnerability as being negatively affected after adaptation measures, which we quantify by multiplying the modeled scenario average of regional adverse impacts with relative indicators of sensitivity and adaptive capacity from regional statistics data. These definitions and calculation methods extend the concepts of vulnerability from climate change adaptation literature^43^. We provide a concept overview of the benefit-vulnerability calculation in Fig. 7, describe our calculation methods in the following section, and provide an example calculation in the Supplementary Methods.

We calculate scenario averages of benefit for each region r and impact type *k* with the Eq. (1):

1 $${B}_{r}^{k}=\frac{1}{N}\mathop{\sum}\limits_{t}^{T}\mathop{\sum}\limits_{n}^{N}{E}_{r,n,t}^{k}$$

where *B* is the average benefit for a specific region r and impact type *k* (e.g., job increases per capita); *E* is the specific benefit (e.g., job increases per capita) associated with a specific region *r*, scenario *n*, and technology *t* (e.g., onshore wind); *N* refers to the total number of MGA scenarios and the minimum cost scenario (*N* = 249) and *T* refers to the total number of technologies for electricity generation, storage, and transmission (*T* = 21). We do not normalize average benefit to keep the original units and values.

In contrast to benefits, regional vulnerability is calculated in two steps. In the first step, we calculate scenario averages of regional vulnerability for each region r and impact type *k* with the Eq. (2):

2 $${V}_{r}^{k}=\frac{1}{N}\mathop{\sum}\limits_{t}^{T}\mathop{\sum}\limits_{n}^{N}{E}_{r,n,t}^{k}\mathop{\sum}\limits_{i}^{I}{{w}_{i}^{k}S}_{r,i}^{k}\left[1-\mathop{\sum}\limits_{j}^{J}{w}_{j}^{k}{A}_{r,j}^{k}\right]$$

where *V* is the average vulnerability for a specific region *r* and impact type *k* (e.g., job losses per capita); *E* is the specific adverse impact (e.g., job losses per capita) associated with a specific region r, scenario *n*, and technology *t* (e.g., onshore wind); *S* is a specific sensitivity indicator with identifier *i* (e.g., long-term unemployment rate); *A* is a specific adaptive capacity indicator with identifier *j* (e.g., unemployment benefits); I refers to the total number of sensitivity indicators per impact type *k* (*I* = 3); *J* refers to the total number of adaptive capacity indicators per impact type *k* (*J* = 3). Equation (2) applies the weighted-sum method of multi-criteria analysis with equal weights *w*^91^. All adverse impact measures *E*, sensitivity indicators *S*, and adaptive capacity indicators *A* are given as normalized values between 0 (low) and 1 (high). In the second step, we normalize the resulting scenario averages of vulnerability to normalized values between 0 (low) and 1 (high) with min-max normalization^90^.

Finally, we quantify composite indices of benefit and vulnerability that account for benefits and vulnerabilities of all six impacts that we asses. These two composite indices are treated separately as benefits and vulnerabilities do not necessarily compensate each other. For example, regions can have both high benefits due to new employment opportunities in renewable electricity sector and high vulnerabilities due to employment losses from fossil fuel industry. In the first step of calculating composite benefits, we first normalize all six benefit indices to values between 0 (low) and 1 (high) with min-max normalization^90^. In the second step, using weighted-sum method of multi-criteria analysis^91^, we add all six normalized benefit indices (i.e., regarding investment, decreased annual average electricity prices, increased employment, decreased greenhouse gas and particulate matter emissions, and decreased land use) with equal weights of 1/6. In the third and final step, we calculate composite benefit indices by normalizing the resulting sum to values between 0 (low) and 1 (high) with min-max normalization^90^. Regarding composite vulnerability indices, we skip the first step as all six individual vulnerability indices are already normalized.

### Reporting summary

Further information on research design is available in the Nature Portfolio Reporting Summary linked to this article.

## Supplementary information

Supplementary Information

Reporting Summary

## Data Availability

All input data of this study are provided in the Supplementary Methods or are openly available on public repositories that we cite. The model output data are publicly available at Zenodo 10.5281/zenodo.7777215. Source data are provided with this paper. Here we list an overview of the databases used on this study: • Data on existing conventional power plants are publicly accessible from Open Power System Data: 10.25832/conventional_power_plants/2020-10-01 • Data on existing renewable (excluding hydro power) are publicly accessible from Open Power System Data: 10.25832/renewable_power_plants/2020-08-25 • Data on existing hydro power plants are publicly accessible from the JRC hydro-power database: 10.5281/zenodo.3862722 • Data on existing and potential transmission infrastructure are publicly accessible from the European PyPSA dataset: 10.5281/zenodo.3886532 • Data on installed generation capacity potentials of wind and solar PV are are publicly accessible from a European modeling study: 10.5281/zenodo.3533038 • Data on installed generation capacity potentials of biomass are publicly accessible from the JRC ENSPRESO database: http://data.europa.eu/89h/74ed5a04-7d74-4807-9eab-b94774309d9f • Data on installed generation capacity potentials of geothermal power are publicly accessible from a European geothermal potential study: 10.5194/gtes-2-55-2014 • Data on capacity factor time series for solar PV, wind, and hydro power are publicly accessible from a European modeling study: 10.5281/zenodo.3949553 • Data on regional statistics that we used to compute indicators of sensitivity and adaptive capacity are publicly accessible on the Eurostat database: https://ec.europa.eu/eurostat/data/database.

## Source data

Source Data

## Code availability

Software to process all modeling results and create all associated figures is publicly available on Zenodo 10.5281/zenodo.7777215. All equations of the EXPANSE model are presented in Supplementary Methods. EXPANSE is written with Python 3.7 and applies the Pyomo optimization toolbox to formulate the optimization problem. Pyomo (Version 6.1.2): https://github.com/Pyomo/pyomo. The optimization uses the Gurobi solver. Gurobi (Version 9.5.0): https://www.gurobi.com. All spatial data analysis is performed with the Geopandas package. Geopandas (Version 0.10.2): https://github.com/geopandas/geopandas. All other data processing is done with the Pandas package. Pandas (Version 1.3.4): https://github.com/pandas-dev/pandas. Background maps were added for visualization purposes by applying the Cartopy package. Cartopy (Version 0.21.1): https://github.com/SciTools/cartopy.
