# Supplementary material for: A low-carbon electricity sector in Europe risks sustaining regional inequalities in benefits and vulnerabilities
Source: Nat Commun. 2023 Apr 18;14:2205. doi: 10.1038/s41467-023-37946-3 (PMC10111333; doi:10.1038/s41467-023-37946-3)
Supplement: Supplementary file 1 — Supplementary Information [file 41467_2023_37946_MOESM1_ESM.pdf]

Supplementary Information for

A low-carbon electricity sector in Europe risks sustaining regional inequalities in benefits and vulnerabilities

Authors: **Jan-Philipp Sasse[1]*, Evelina Trutnevyte[1]**

[1] Renewable Energy Systems, Institute for Environmental Sciences (ISE), Section of Earth and Environmental Sciences, University of Geneva, Uni Carl Vogt, Boulevard Carl Vogt 66, CH-1211 Geneva 4, Switzerland

* corresponding author (jan-philipp.sasse@unige.ch)

1. Supplementary Methods

1.1. Impact factors to evaluate regional impacts in EXPANSE

Regional impacts regarding jobs, greenhouse gas emissions, particulate matter emissions, and land use are calculated by applying corresponding impact factors on installed capacity and electricity generation (shown in Supplementary Table 1). Regional direct employment accounts for jobs in construction, installation, operation, maintenance, and decommissioning, but not in manufacturing, fuel extraction (e.g., coal mining), and transport. Employment in construction, installation, and decommissioning are given in job-years per MW, while employment in operation and maintenance are given in jobs per MW. We calculate regional impact factors for employment by first harmonizing all units of impact factors to jobs per MW and then adding all harmonized impact factors. Greenhouse gas and particulate matter emissions are direct emissions from fuel combustion for electricity generation. We assume that not all greenhouse gas emissions from combustion of biomass were previously absorbed from the atmosphere during biomass growth. These low values of greenhouse gas emissions for biomass are within uncertainty ranges of other studies[1]. Direct land use refers to the industrial area and artificial lake area occupied by power plants and storage plants. We exclude land used for fuel extraction and transport.

Supplementary Table 1: Impact factors of electricity generation, storage, and transmission in 2035. Percentages in parentheses next to employment factors of electricity generation and storage indicate shares of included jobs compared to all jobs in manufacturing, construction, installation, operation, maintenance, decommissioning, fuel extraction and transport.

Technology	Regional direct employment (Jobs MW^{-1})	Direct greenhouse gas emissions (tCO_{2-eq} MWh_{el}^{-1})	Direct particulate matter emissions ($kgPM_{10}$ MWh_{el}^{-1})	Direct land use (m^2 MW^{-1})
Wind – onshore	0.53 (74%) [2]	-	-	1087.0 [3]
Wind – offshore	1.07 (63%) [2]	-	-	-
Solar PV – open field	1.25 (82%) [2]	-	-	7491.2 [3]
Solar PV – rooftop	1.27 (83%) [2]	-	-	-
Large hydro dams	0.32 (85%) [2]	-	-	11691.7 [3]
Large hydro run of river	0.32 (85%) [2]	-	-	182.3 [3]
Small hydropower	0.32 (85%) [2]	-	-	182.3 [3]
Biogas	2.22 (56%) [2]	0.045 [4]	0.200 [5]	3000.0 [3]
Woody biomass	2.07 (52%) [2]	0.045 [4]	0.334 [5]	1250.0 [3]
Waste	2.07 (48%) [2]	0.106 [4]	0.100 [5]	691.8 [3]
Geothermal	0.63 (83%) [2]	-	-	2500.0 [3]
Nuclear	0.80 (97%) [2]	-	-	100.0 [3]
Hard coal	0.46 (15%) [2]	0.840 [4]	0.206 [5]	110.0 [3]
Lignite	0.46 (13%) [2]	0.910 [4]	0.320 [5]	115.1 [3]
Gas	0.19 (36%) [2]	0.350 [4]	0.101 [5]	240.0 [3]
Oil	0.18 (28%) [2]	0.708 [6]	0.200 [5]	250.0 [3]
Pumped hydro storage	1.15 (95%) [7]	-	-	61000.1 [3]
Battery storage	0.35 (21%) [7]	-	-	15.0 [8]
Hydrogen storage	0.63 (86%) [7]	-	-	80.0 [9]
Transmission	180.00 jobs $TWkm^{-1}$ [10]	-	-	-

1.2. Input data and assumptions for the EXPANSE model

We collect data on existing infrastructure in Europe for electricity generation[11–14], storage[13], and transmission[15]. We amend the spatial representation of existing electricity generation infrastructure for biomass (biogas, woody biomass, and waste), solar PV (open-field and rooftop), and wind (onshore and offshore) with global[16], continental[17], and national data[18–28].

We collect data on installed generation capacity potentials[29–32] for biomass, geothermal, hydropower, solar PV, and wind from other modeling studies. These potentials only consider areas that are eligible for renewable electricity generation based on multiple constraints (e.g., land cover and use, existing settlements, land slope, environmentally protected areas, and water depths) and land requirements of generation technologies. In order to account for maximum installed capacity growth rates until 2035, we limit solar PV and wind generation capacities to maximum country-specific estimates from European Transmission System Operators[33]. Similarly, we limit woody biomass generation capacities to 20% of the technical potentials[30]. Likewise, we limit the technical potential of geothermal generation capacities that we collect from a European geothermal modeling study[31] to areas where the levelized cost of electricity generation (LCOE) is below 150 EUR MWh^{-1} and assume that no more than 1% of this economic potential can be used in 2035. Setting these two parameters of maximum LCOE and capacity deployment reflect the maximum capacity estimates of European Transmission System Operators[33].

We collect data on capacity factor time series for solar PV, wind, and hydropower from a recent modeling study[34]. EXPANSE uses capacity factor time series to calculate the maximum electricity that can be generated in each time step per unit of installed capacity. Capacity factor time series for wind and solar PV use bias-corrected data from Renewables Ninja[35]. Capacity factor time series for hydropower are calculated in two steps. In the first step, water inflow time series are calculated based on ERA5 runoff data[36] and hydrological basins[37]. In the second step, bias-correction factors are derived from national generation data from IRENA[38] and then applied to the water inflow time series to represent the actual inflow available for electricity generation.

We define that no new nuclear and fossil fuel (hard coal, lignite, gas, and oil) generation capacities can be built, except for currently planned expansions that are documented in the National Energy and Climate Plans of EU member countries[39]. We assume that all nuclear generation capacities are decommissioned in Belgium and Germany in 2035.

We assume that existing storage capacities of large hydropower dams and pumped hydro storage[13,40] will not change until 2035. We apply a greenfield approach for battery and hydrogen storage with initially zero storage capacities in all regions. EXPANSE can extend these storage capacities, depending on the storage requirements of each scenario. Hydrogen storage is modeled as a stationary storage, like battery, and we assume that hydrogen cannot be transported between or within countries.

We collect data on transmission line capacities from the European PyPSA dataset for our transmission grid layout of 128 grid nodes[15]. This dataset accounts for both currently

existing and new transmission lines that are at least in the permitting phase. We assume that currently existing transmission line capacities can be extended by the EXPANSE model to up to two times their current capacity, but not reduced. To account for grid security, we limit hourly power flows over transmission lines to 50% of their nominal capacity. If that limit is reached, EXPANSE extends the transmission line capacity.

We estimate hourly electricity demand in 2035 at each grid node. We do this by first collecting country-level hourly electricity demand in 2030 from estimated national trends of European Transmission System Operators[33]. These demand estimates capture changes in hourly electricity demand from residential, tertiary, and industry sectors, as well as from electrification of transport and heating. Next, we spatially disaggregate this country-level demand to each grid node by applying spatial weights. We derive these spatial weights from the annual demand at each grid node of the European PyPSA model[15], which is based on the regional distribution of population and gross domestic product. An overview of national estimates for electricity demand in 2035 and actual demand in 2020 is shown in Supplementary Table 2.

Supplementary Table 2: Overview of national electricity demand in 2020 and 2035. Table shows annual values for national electricity demand. We refer to national electricity demand as the total load, which is defined as the sum of net electricity demand and transmission and distribution losses. Electricity demand data of 2020 is collected from Eurostat[41] and the Swiss Federal Office of Energy[42].

Country	2020 (TWh)	2035 (TWh)	Change (%)	Country	2020 (TWh)	2035 (TWh)	Change (%)
Albania	7.6	9.9	30	Italy	301.8	324.4	7
Austria	65.6	78.8	20	Lithuania	12.1	14.2	17
Bosnia Herzegovina	11.0	12.9	17	Luxembourg	6.3	9.5	51
Belgium	83.3	91.3	10	Latvia	7.1	8.0	13
Bulgaria	33.1	39.2	18	Montenegro	3.1	4.4	42
Switzerland	59.9	61.0	2	North Macedonia	7.4	9.1	23
Czech Republic	63.1	77.8	23	Netherlands	116.4	118.8	2
Germany	525.5	550.7	5	Norway	131.2	148.7	13
Denmark	33.3	45.8	38	Poland	156.3	182.2	17
Estonia	8.7	8.8	1	Portugal	52.5	57.7	10
Greece	52.1	54.4	4	Romania	52.9	63.9	21
Spain	252.5	276.9	10	Serbia	32.6	46.2	42
Finland	81.2	97.3	20	Sweden	133.0	152.7	15
France	458.8	485.9	6	Slovenia	13.9	16.6	19
Croatia	17.3	17.2	-1	Slovak Republic	26.4	35.6	35
Hungary	44.5	42.8	-4	United Kingdom	302.0	317.5	5
Ireland	30.8	41.1	33	Total	3183.0	3501.3	10

The EXPANSE model considers annualized capital and variable costs to optimize electricity generation, storage, and transmission capacity. These costs are estimated with techno-economic parameters for electricity generation (Supplementary Table 3), storage (Supplementary Table 4), and transmission (Supplementary Table 5). These cost assumptions are long-term estimates for the year 2035 and include changes in costs from technological learning. Prices of natural gas and other fossil fuels in 2035 are from the IEA's World Energy Outlook[43], which we consider the best available long-term estimates. We define total system costs as the sum of annualized capital and variable costs from electricity generation, storage, and transmission. We assume a weighted average cost of capital (WACC) of 3% for all technologies and regions. We do not include any subsidies or taxes (e.g., feed-in tariffs or carbon tax).

Supplementary Table 3: Techno-economic parameters of electricity generation in 2035. CAPEX – capital expenditures, FOM – fixed operation costs, VOM – variable operation costs. EXPANSE applies minimum hourly capacity factors to account for flexibility constraints of electricity generation from biomass, fossil fuel, geothermal, and nuclear power plants.

**Technology**	**CAPEX (EUR kW^{-1})**	**FOM (% of CAPEX $year^{-1}$)**	**VOM (EUR MWh_{el}^{-1})**	**Efficiency (%)**	**Fuel costs (EUR MWh_{el}^{-1})**	**Lifetime (years)**	**Minimum hourly capacity factor (%)**
Wind – onshore	1250 [44]	2.1 [44]	1.3 [44]	100.0 [45]	-	25 [46]	-
Wind – offshore	1870 [44]	2.5 [44]	2.6 [44]	100.0 [45]	-	25 [46]	-
Solar PV – open field	350 [44]	1.9 [44]	0.01 [44]	100.0 [45]	-	25 [46]	-
Solar PV – rooftop	870 [44]	1.2 [44]	0.01 [44]	100.0 [45]	-	25 [46]	-
Large hydro dams	2200 [4]	1.5 [4]	1.0 [4]	90.0 [47]	-	60 [4]	-
Large hydro run of river	3000 [47]	1.5 [4]	1.0 [4]	90.0 [47]	-	60 [4]	-
Small hydropower	5620 [4]	1.5 [4]	1.0 [4]	90.0 [47]	-	60 [4]	-
Biogas	2640 [4]	4.1 [4]	3.1 [4]	41.0 [4]	31.0 [47]	20 [4]	40 [48]
Woody biomass	2260 [4]	2.2 [4]	3.5 [4]	37.0 [4]	45.0 [47]	25 [4]	40 [48]
Waste	5055 [4]	3.0 [4]	6.9 [4]	35.5 [4]	6.0 [47]	25 [4]	40 [48]
Geothermal	8800 [4]	1.9 [4]	0.0 [4]	12.0 [4]	-	30 [4]	40 [48]
Nuclear	3950 [4]	1.8 [4]	2.5 [4]	38.0 [4]	7.9 [47]	60 [4]	60 [48]
Hard coal	1600 [4]	2.5 [4]	3.6 [4]	42.5 [45]	15.6 [43]	40 [4]	40 [48]
Lignite	2000 [4]	2.5 [4]	4.5 [4]	37.5 [45]	6.1 [43]	40 [4]	40 [48]
Gas	800 [47]	2.5 [47]	4.0 [47]	58.5 [45]	41.5 [43]	30 [47]	40 [48]
Oil	400 [47]	1.5 [47]	3.0 [47]	35.0 [45]	136.8 [43]	40 [47]	40 [48]

Supplementary Table 4: Techno-economic parameters of electricity storage in 2035. CAPEX – capital expenditures, FOM – fixed operation costs, VOM – variable operation costs. Capital expenditures are annualized and consider specific power and energy costs as well as replacement costs. Efficiencies are shown as round-trip efficiencies. Maximum energy capacities of storage units are directly linked to the maximum power capacities of storage units with the discharge time variable. Discharge time refers to the number of hours needed to discharge the storage unit at maximum power[49]. Hours per discharge of pumped hydro storage is location-specific and varies between indicated values. These values are derived from installed energy and power capacities of existing pumped hydro units[40].

Technology	CAPEX (EUR kW^{-1} $year^{-1}$)	Discharge time (h)	FOM (% of CAPEX)	VOM (EUR MWh_{el}^{-1})	Efficiency (%)	Lifetime (years)	Extendable capacity
Pumped hydro storage	168 [49]	0.1–362.8 [40]	0.7 [49]	1 [49]	0.75 [49]	55 [49]	No
Battery storage	135 [49]	6 [49]	1.5 [49]	3 [49]	0.81 [49]	13 [49]	Yes
Hydrogen storage	302 [49]	168 [49]	0.9 [49]	0 [49]	0.46 [49]	18 [49]	Yes

Supplementary Table 5: Techno-economic parameters of electricity transmission in 2035. CAPEX – capital expenditures, FOM – fixed operation costs, VOM – variable operation costs, HVDC – high voltage direct current transmission line, HVAC – high voltage alternating current transmission line.

Technology	CAPEX (EUR MW^{-1} km^{-1})	FOM (% of CAPEX $year^{-1}$)	VOM (EUR MWh_{el}^{-1})	Efficiency (%)	Lifetime (years)
HVAC overhead	400 [50]	2 [50]	2 [4]	100 [50]	40 [50]
HVDC overhead	400 [50]	2 [50]	2 [4]	100 [50]	40 [50]
HVDC submarine	2000 [50]	2 [50]	2 [4]	100 [50]	40 [50]

We define a maximum limit on total greenhouse gas emissions in 2035 so that modeled scenarios are consistent with emission reductions of net zero target in 2050, called for the European Green Deal[51]. This implies around 70% reductions in electricity-related greenhouse gas emissions by 2035 as compared to 2019[52,53]. Thus, we enforce a maximum limit of 245 $MtCO_{2\text{-}eq}$ $year^{-1}$ in total greenhouse gas emissions for all low-carbon MGA and minimum cost scenarios. This maximum emissions limit does not apply to the frozen scenario that represents a continuation of the current electricity system.

1.3. Sensitivity and adaptive capacity indicators

Supplementary Table 6 and Supplementary Table 7 provide detailed descriptions of sensitivity and adaptive capacity indicators. All indicators are spatially-explicit and are derived from regional statistics data.

Supplementary Table 6: Sensitivity indicators. Min-max normalized[54] values are shown in parentheses. Variable *w* refers to weights for Equation (2) in the main manuscript. PPS – purchasing power standard. NUTS – Nomenclature of Territorial Units for Statistics[55].

Vulnerability	Sensitivity indicator	w	Average	Standard deviation	Min	Max	Unit	Year	NUTS level
Divestment	Share of total labor costs of electricity supply sector (NACE D35) as compared to all industry, construction, and services sectors (NACE B–S)[56]. Includes wages, salaries, direct remuneration, bonuses, allowances, and employers' social security contributions.	1/3	1.8 (0.20)	1.5 (0.18)	0.0 (0.00)	8.6 (1.00)	%	2016	1
Divestment	General government consolidated gross debt as % of GDP[57]. Includes central government, state government, local government, and social security funds.	1/3	75.4 (0.39)	36.8 (0.21)	8.4 (0.00)	180.5 (1.00)	%	2019	0
Divestment	Gross domestic product (GDP)[58]. GDP is an inverted indicator so that high GDP indicates low sensitivity.	1/3	30207 (0.88)	15618 (0.09)	7500 (1.00)	190500 (0.00)	PPS capita^{-1}	2018	2
Increased electricity prices	Share of population with arrears on utility bills for the main dwelling within the last 12 months[59].	1/3	7.83 (0.18)	8.72 (0.25)	1.50 (0.00)	36.90 (1.00)	%	2019	0
Increased electricity prices	Share of private household consumption expenditure for electricity[60].	1/3	2.95 (0.21)	1.20 (0.17)	1.40 (0.00)	8.70 (1.00)	%	2015	0
Increased electricity prices	Share of the population at risk of poverty or social exclusion[61]. The at-risk-of-poverty threshold is set at 60% of the median equivalized disposable income.	1/3	21.76 (0.30)	8.44 (0.18)	7.90 (0.00)	53.60 (1.00)	%	2019	2
Employment losses	Long-term (for at least 12 months) unemployment rate[62].	1/3	2.9 (0.14)	3.7 (0.20)	0.3 (0.00)	18.5 (1.00)	%	2019	2
Employment losses	Jobs in electricity supply sector (NACE D35) per 100'000 inhabitants[63].	1/3	2.5 (0.18)	1.8 (0.13)	0.0 (0.00)	14.2 (1.00)	Jobs capita^{-1}	2018	2
Employment losses	Share of population living in households with very low work intensity[64].	1/3	8.6 (0.29)	3.6 (0.16)	2.0 (0.00)	25.0 (1.00)	%	2019	2
Increased greenhouse gas emissions	Average cooling degree days (CDD) per year between 1990 and 2015[65].	1/3	79.6 (0.17)	107.8 (0.23)	0.0 (0.00)	475.1 (1.00)	CDD	2019	3
Increased greenhouse gas emissions	Cumulative fatalities between 1990 and 2016 due to extremely high temperatures per million inhabitants[65].	1/3	117.2 (0.32)	131.4 (0.36)	0.0 (0.00)	360.6 (1.00)	Fatalities capita^{-1}	2016	0
Increased greenhouse gas emissions	Cumulative economic losses between 1980 and 2019 due to extreme weather and climate related events[66].	1/3	946.0 (0.53)	354.1 (0.23)	119.3 (0.00)	1692.7 (1.00)	PPS capita^{-1}	2019	0
Increased particulate matter emissions	Share of population with self-reported unmet needs for medical and dental care[67].	1/3	4.3 (0.23)	3.5 (0.20)	0.3 (0.00)	17.5 (1.00)	%	2019	2
Increased particulate matter emissions	Share of population with self-perceived bad or very bad health[68].	1/3	8.5 (0.38)	2.5 (0.19)	3.3 (0.00)	16.8 (1.00)	%	2019	0
Increased particulate matter emissions	Years of life lost (YLL) attributable to particulate matter emissions per 100'000 inhabitants[65].	1/3	808.8 (0.32)	372.2 (0.22)	249.7 (0.00)	1971.6 (1.00)	YLL capita^{-1}	2015	0
Increased land use	Population density in persons per km^2 of total area[69].	1/3	462.8 (0.04)	1254.4 (0.11)	3.4 (0.00)	11509.1 (1.00)	person km^{-2}	2018	2
Increased land use	Number of arrivals at tourist accommodation establishments per inhabitant[70].	1/3	1.5 (0.21)	1.1 (0.17)	0.1 (0.00)	6.6 (1.00)	arrival capita^{-1}	2019	2
Increased land use	Utilized agricultural area as % of total area[71].	1/3	42.6 (0.46)	19.2 (0.21)	0.0 (0.00)	93.1 (1.00)	%	2013	2

Supplementary Table 7: Adaptive capacity indicators. Min-max-normalized[54] values are shown in parentheses. Variable w refers to weights for Equation (2) in the main manuscript. PPS – purchasing power standard. NUTS – Nomenclature of Territorial Units for Statistics[55].

Vulnerability	Adaptive capacity indicator	w	Average	Standard deviation	Min	Max	Unit	Year	NUTS level
Divestment	Share of employment in high-tech sectors[72]. Includes high-tech manufacturing and knowledge-intensive services.	1/3	3.8 (0.29)	2.1 (0.18)	0.5 (0.00)	11.9 (1.00)	%	2019	2
Divestment	Motorway density in km per 1'000 km^2 of total area[73].	1/3	28.0 (0.15)	25.7 (0.13)	0.0 (0.00)	191.0 (1.00)	km km^{-2}	2019	2
Divestment	Government quality index[74]. Multi-dimensional measure of impartiality, quality of public services, and corruption.	1/3	0.2 (0.54)	1.1 (0.24)	-2.3 (0.00)	2.3 (1.00)	index	2017	2
Increased electricity prices	Housing benefits to help households meet the cost of housing[75]. Includes cash transfers for rent assistance, social housing, and owner-occupier benefits.	1/3	132.7 (0.39)	124.8 (0.36)	0.0 (0.00)	343.2 (1.00)	PPS $capita^{-1}$	2018	0
Increased electricity prices	Net disposable income (i.e., income after subtracting income taxes and pension contributions)[76].	1/3	16646.8 (0.44)	4500.8 (0.20)	6700.0 (0.00)	29362.0 (1.00)	PPS $capita^{-1}$	2018	2
Increased electricity prices	Share of near-zero energy buildings in new construction for residential buildings[77]. The low energy demand of these buildings is mostly covered by renewable sources produced on-site or nearby.	1/3	0.2 (0.23)	0.1 (0.16)	0.1 (0.00)	0.8 (1.00)	%	2016	0
Employment losses	Unemployment benefits[75]. Mostly includes cash benefits for unemployment and early retirement due to labor market reasons.	1/3	299.2 (0.35)	203.3 (0.25)	11.7 (0.00)	838.2 (1.00)	PPS $capita^{-1}$	2018	0
Employment losses	Expenditure for labor market policy (LMP) interventions[78]. Includes LMP services, LMP measures (e.g., job training), and LMP supports (e.g., financial support during job-search).	1/3	412.9 (0.38)	284.1 (0.27)	10.6 (0.00)	1081.8 (1.00)	PPS $capita^{-1}$	2018	0
Employment losses	Economic activity rate[79]. Defined as share of economically active population (both employed and unemployed).	1/3	65.4 (0.58)	5.8 (0.17)	45.2 (0.00)	79.8 (1.00)	%	2019	2
Increased greenhouse gas emissions	Share of non-vulnerable population[80]. Non-vulnerable population are people with ages between 5 and 75 years old.	1/3	85.6 (0.56)	1.7 (0.19)	80.5 (0.00)	89.7 (1.00)	%	2019	2
Increased greenhouse gas emissions	Share of dwellings equipped with air conditioning[81]. Does not include fans.	1/3	8.5 (0.18)	11.4 (0.25)	0.5 (0.00)	45.3 (1.00)	%	2007	0
Increased greenhouse gas emissions	Insured economic losses as a share of total losses from extreme weather events between 1980 and 2019[66].	1/3	34.1 (0.48)	24.5 (0.36)	1.0 (0.00)	70.0 (1.00)	%	2019	0
Increased particulate matter emissions	Sickness and healthcare benefits[75]. Mostly includes medical care services and goods, but also paid sick leave and other benefits.	1/3	2323.9 (0.54)	1028.9 (0.30)	474.2 (0.00)	3931.2 (1.00)	PPS $capita^{-1}$	2018	0
Increased particulate matter emissions	Available beds in hospitals per 100'000 inhabitants[82].	1/3	489.6 (0.30)	219.1 (0.19)	155.5 (0.00)	1285.3 (1.00)	Beds $capita^{-1}$	2018	2
Increased particulate matter emissions	Life expectancy[83].	1/3	80.9 (0.39)	2.5 (0.21)	73.6 (0.00)	85.5 (1.00)	years	2019	2
Increased land use	Share of land not covered by artificial land[84]. Excludes all land classified as artificial surfaces (grid codes 1–11), such as urban, industrial, and commercial areas.	1/3	87.0 (0.87)	19.3 (0.19)	0.0 (0.00)	99.7 (1.00)	%	2018	2
Increased land use	Share of population not employed in tourism (NACE I55)[63].	1/3	99.2 (0.96)	1.3 (0.08)	82.3 (0.00)	99.9 (1.00)	%	2018	2
Increased land use	Gross value added (GVA) by non-agricultural sectors as a share of GVA from all sectors[85].	1/3	97.6 (0.91)	3.2 (0.13)	74.2 (0.00)	100.0 (1.00)	%	2018	2

1.4. Mathematical formulation of the EXPANSE model

Nomenclature

f_{cost}	total system costs (in EUR year^{-1});
f_{GHG}	total greenhouse gas emissions (in MtCO$_{2\text{-eq}}$ year^{-1});
f_{PM10}	total particulate matter emissions (in ktPM$_{10}$ year^{-1});
f_{LandUse}	total land use (in km^2);
f_{Jobs}	total employment (in jobs);
C	annualized investment costs (in EUR MW^{-1} year^{-1});
O	marginal costs (in EUR MWh^{-1});
G	electricity generation capacity (in MW);
G^{max}	maximum potential electricity generation capacity (in MW);
G^{min}	minimum potential electricity generation capacity (in MW);
g	electricity generation operation (in MWh);
$\tilde{g}^{\mathrm{max}}$	maximum potential capacity factor for electricity generation (per unit of installed capacity);
$\tilde{g}^{\mathrm{min}}$	minimum potential capacity factor for electricity generation (per unit of installed capacity);
H	storage capacity (in MW);
H^{max}	maximum potential storage capacity (in MW);
H^{min}	minimum potential storage capacity (in MW);
E^{SOC}	storage stage of charge (in MWh);
$h^{\mathrm{discharge}}$	storage discharging operation (in MWh);
h^{charge}	storage charging operation (in MWh);
d	maximum hours per discharge (in hours);
e^{inflow}	inflow to the state of charge (in MWh);
e^{spillage}	spillage out of the state of charge (in MWh);
η^{charge}	efficiency of charging storage units;
$\eta^{\mathrm{discharge}}$	efficiency of discharging storage units;
F	transmission capacity (in MW);
F^{max}	maximum potential transmission capacity (in MW);

$F^{\min}$	minimum potential transmission capacity (in MW);
f	transmission flow (in MWh);
L	length of transmission line (in km);
K	incidence matrix of transmission grid network;
D	exogenous inelastic electricity demand (in MWh);
r	NUTS-2 region label;
m	NUTS-2 regions connected to specific transmission grid node;
n	transmission grid node label;
l	transmission line label;
c	technology type label;
t	time step label;
w	constant weight (equal to six when modeling six-hour time steps);
i^{GHG}	impact factor for greenhouse gas emissions (see Supplementary Table 1);
i^{PM10}	impact factor for particulate matter emissions (see Supplementary Table 1);
i^{LandUse}	impact factor for land use (see Supplementary Table 1);
i^{Jobs}	impact factor for employment (see Supplementary Table 1);
C_{slack}	moving slack[86], varies between 0% and 20%;
α	randomly drawn number from a uniformly distributed set {-1,0,1};

Objective function of cost minimization mode

The basic version of the EXPANSE model is a cost optimization model, which minimizes total system costs $\mathrm{f}_{\mathrm{cost}}$ across all NUTS regions r, transmission grid nodes n, transmission lines l, technology types c, and time steps t.

$$f_{\mathrm{cost}} = \sum_{r,c} C_{r,c} G_{r,c} + \sum_{n,c} C_{n,c} H_{n,c} + \sum_{l} C_l F_l + \sum_{t} w_t \left[\sum_{r,c} O_{r,c,t} g_{r,c,t} + \sum_{n,c} O_{n,c,t} h_{n,c,t}^{\mathrm{discharge}} + \sum_{l} O_{l,t} \left| f_{l,t} \right| \right] \quad (1)$$

Total system costs include annualized investment costs C (in EUR MW^{-1} year^{-1}) and marginal costs O (in EUR MWh^{-1}) of electricity generation, storage, and transmission infrastructure. We define annualized investment costs as the sum of annualized capital

expenditures (CAPEX) and annual fixed operation costs (FOM). We define annualized marginal costs as the sum of annual variable operation costs (VOM) and fuel costs.

Electricity generation capacities G (in MW) and operation g (in MWh) are spatially represented at each of the 296 NUTS regions r. Storage capacities H (in MW) and charging and discharging operation h (in MWh) are spatially represented at each of the 128 transmission grid nodes n. Transmission line capacities F (in MW) and transmission flows f (in MWh) between grid nodes are spatially represented at each transmission line l.

Supplementary Equation (1) applies constant weights w to annualize all marginal costs so that the sum of weights equals 8'760. For example, if EXPANSE is configured to a temporal resolution of six-hour time steps, the weights are set to 6.

Supplementary Equation (1) contains an absolute term for the transmission operation costs of flows f, which makes the equation non-linear. This absolute term ensures that transmission operation costs are always positive and do not reduce total system costs if flows are negative (i.e., flowing in the opposite direction). We linearize Supplementary Equation (1) by applying a linear problem transformation technique for absolute values[87] and by replacing it with the equivalent Supplementary Equations (2)-(4).

$$f_{\text{cost}} = \sum_{r,c} C_{r,c} G_{r,c} + \sum_{n,c} C_{n,c} H_{n,c} + \sum_{l} C_l F_l + \sum_t w_t \left[\sum_{r,c} O_{r,c,t} g_{r,c,t} + \sum_{n,c} O_{n,c,t} h_{n,c,t}^{\text{discharge}} + \sum_l O_{l,t} f_{l,t}^{+} \right] \quad (2)$$

$$f_{l,t}^{+} \geq f_{l,t} \quad (3)$$

$$f_{l,t}^{+} \geq -f_{l,t} \quad (4)$$

Objective function of MGA mode

After the initial run of EXPANSE in a cost minimization mode (Supplementary Equation (1)), EXPANSE then uses an adapted MGA algorithm to calculate additional scenarios that simultaneously minimize or nearly minimize total system costs, greenhouse gas emissions, particulate matter emissions, land use, and maximizes total jobs. Costs of these MGA scenarios are configured to not exceed 20% of the costs of the minimum cost scenario[88]. In addition, total greenhouse gas emissions are configured to not exceed 245 $MtCO_{2\text{-eq}}$ $year^{-1}$. All MGA scenarios are computer-generated by EXPANSE with the following steps:

1. Solve the Pareto frontiers to obtain the first set of 48 MGA scenarios.

EXPANSE calculates the Pareto frontiers between minimizing total system costs and the other four objectives of minimizing total greenhouse gas emissions f_{GHG} (in $MtCO_{2\text{-eq}}$ $year^{-1}$), total particulate matter emissions f_{PM10} (in $ktPM_{10}$ $year^{-1}$), and total land use f_{LandUse} (in km^2), and maximizing total employment f_{Jobs}.

$$f_{\text{GHG}} = \sum_{r,c,t} w_t g_{r,c,t} i_c^{\text{GHG}} \quad (5)$$

$$f_{\text{PM10}} = \sum_{r,c,t} w_t g_{r,c,t} i_c^{\text{PM10}} \quad (6)$$

$$f_{\text{LandUse}} = \sum_{r,c} G_{r,c} i_c^{\text{LandUse}} + \sum_{n,c} H_{n,c} i_c^{\text{LandUse}} \quad (7)$$

$$f_{\text{Jobs}} = \sum_{r,c} G_{r,c} i_c^{\text{Jobs}} + \sum_{n,c} H_{n,c} i_c^{\text{Jobs}} + \sum_{l} F_l L_l i_c^{\text{Jobs}} \quad (8)$$

where i_c^{GHG} (in $tCO_{2\text{-eq}}$ MWh_{el}^{-1}), i_c^{PM10} (in $kgPM_{10}$ MWh_{el}^{-1}), i_c^{LandUse} (in m^2 MW^{-1}), and i_c^{Jobs} (in Jobs MW^{-1}) are the corresponding impact factors listed in Supplementary Table 1, and L_l (in km) are the lengths of transmission lines.

EXPANSE calculates these Pareto frontiers for pre-defined slacks C_{slack} with values of {0.5%, 1%, 2%, 3%, 4%, 5%, 7.5%, 10%, 12.5%, 15%, 17.5%, 20.0%}.

$$f_{\text{cost}} \leq (1 + C_{\text{slack}}) f_{\text{cost}}^{\min} \quad (9)$$

Solving these four objectives for 12 pre-defined slacks results in the first set of 48 MGA scenarios.

2. Solve additional MGA scenarios.

Next, each additional MGA scenario is computer-generated by first obtaining a randomly drawn cost slack from a uniform distribution (e.g., 4.2%).

$$0\% \leq C_{\text{slack}} \leq 20\% \quad (10)$$

EXPANSE then applies computer-generated random objective functions for investment variables (i.e., country and technology-specific generation capacities $G_{r,c}$). $\alpha_{r,c}$ are randomly drawn numbers from a uniformly distributed set {-1,0,1} for each NUTS region r and generation technology type c.

$$\max_{G} \sum_{r,c} \alpha_{r,c} G_{r,c} \quad (11)$$

EXPANSE ensures the spread and diversity of MGA scenarios not only in terms of costs, but also in terms of other impacts. EXPANSE does this by additionally applying computer-

generated random constraints on greenhouse gas emissions, particulate matter emissions, land use, and employment (indicated by an asterisk in Supplementary Equations (12)-(15)).

$$f_{\mathrm{GHG}} \leq f_{\mathrm{GHG}}^{*} \qquad (12)$$
$$f_{\mathrm{PM10}} \leq f_{\mathrm{PM10}}^{*} \qquad (13)$$
$$f_{\mathrm{Jobs}} \geq f_{\mathrm{Jobs}}^{*} \qquad (14)$$
$$f_{\mathrm{LandUse}} \leq f_{\mathrm{LandUse}}^{*} \qquad (15)$$

3. Iterate step 2 until a wanted number of additional MGA scenarios are found.

Generator constraints

Electricity generation capacities $\mathrm{G_{r,c}}$ (in MW) are limited by minimum and maximum values for each NUTS region r and technology type c.

$$G_{r,c}^{\min} \leq G_{r,c} \leq G_{r,c}^{\max} \qquad \forall r, c \qquad (16)$$

Electricity generation operation $\mathrm{g_{r,c,t}}$ (in MWh) is constrained by maximum capacity factors $\tilde{\mathrm{g}}_{\mathrm{r,c,t}}^{\max}$ of each generator for each NUTS region r, technology type c, and time step t. Maximum capacity factors are given in per unit of generation capacity (e.g., 80% of installed capacity). Maximum capacity factors are time-dependent for solar, wind, and hydro power and otherwise constant at 100% for all other generators.

$$g_{r,c,t} \leq \tilde{g}_{r,c,t}^{\max} G_{r,c} \qquad \forall r, c, t \qquad (17)$$

In addition, electricity generation operation is constrained by minimum capacity factors $\tilde{\mathrm{g}}_{\mathrm{c}}^{\min}$ to account for the inflexibility of thermal generation units. For example, we assume that nuclear power plants cannot operate at less than 60% of their installed capacity. Minimum capacity factors are not time or region-specific and only vary for each technology type c (see Supplementary Table 3).

$$g_{r,c,t} \geq \tilde{g}_{c}^{\min} G_{r,c} \qquad \forall r, c, t \qquad (18)$$

Storage constraints

Storage capacities $\mathrm{H_{n,c}}$ (in MW) are constrained by maximum and minimum values for each grid node n and technology type c.

$$H_{n,c}^{\min} \leq H_{n,c} \leq H_{n,c}^{\max} \qquad \forall n, c \qquad (19)$$

Storage charge operation $\mathrm{h_{n,c,t}^{charge}}$ (i.e., when it increases the state of charge) and storage discharge operation $\mathrm{h_{n,c,t}^{discharge}}$ (i.e., when it decreases the state of charge) are constrained by the storage capacities $\mathrm{H_{n,c}}$ for each grid node n, technology type c, and time step t.

$$0 \leq h_{n,c,t}^{\mathrm{charge}} \leq H_{n,c} \qquad \forall n, c, t \qquad (20)$$

$$0 \leq h_{n,c,t}^{\text{discharge}} \leq H_{n,c} \qquad \forall n,c,t \qquad (21)$$

Storage state of charge is given by the variable $E_{n,c,t}^{SOC}$ (in MWh) and is constrained by the storage capacities $H_{n,c}$ (in MW) and the maximum discharge hours $d_{n,c}$ (in hours) of each storage unit. This constraint is applied for each transmission grid node n, technology type c, and time step t. Maximum discharge hours $d_{n,c}$ per technology type are listed in Supplementary Table 4. Maximum storage state of charge $E_{n,c}^{SOC,max}$ is not independently optimized but rather directly linked with the storage capacities $H_{n,c}$ and maximum discharge hours $d_{n,c}$ of each storage unit.

$$0 \leq E_{n,c,t}^{\text{SOC}} \leq E_{n,c}^{\text{SOC,max}} = d_{n,c} H_{n,c} \qquad \forall n,c,t \qquad (22)$$

Storage state of charge $E_{n,c,t}^{SOC}$ is related with the storage state of charge of the previous time step $E_{n,c,t-1}^{SOC}$ as well as the storage charge, discharge, inflow, and spillage variables.

$$E_{n,c,t}^{\text{SOC}} = E_{n,c,t-1}^{\text{SOC}} + w_t \left[\eta_c^{\text{charge}} h_{n,c,t}^{\text{charge}} - \frac{1}{\eta_c^{\text{discharge}}} h_{n,c,t}^{\text{discharge}} + e_{n,c,t}^{\text{inflow}} - e_{n,c,t}^{\text{spillage}} \right] \qquad \forall n,c,t \qquad (23)$$

η_c^{charge} and $\eta_c^{discharge}$ are the efficiencies for charging and discharging the storage units. $e_{n,c,t}^{inflow}$ (in MWh) is the inflow to the state of charge (i.e., river inflow into large hydro dams). $e_{n,c,t}^{spillage}$ (in MWh) is the spillage out of the state of charge (i.e., river inflow which is not converted into electricity by large hydro dams).

All storage units are cyclic, which means that they have the same state of charge in the first and last time step of the optimization.

$$E_{n,c,t=0}^{\text{SOC}} = E_{n,c,t=T}^{\text{SOC}} \qquad \forall n,c \qquad (24)$$

Transmission constraints

Transmission line capacities F_l (in MW) are constrained by minimum and maximum values.

$$F_l^{\min} \leq F_l \leq F_l^{\max} \qquad \forall l \qquad (25)$$

Transmission line flows $f_{l,t}$ (in MWh) are constrained by transmission line capacities F_l and a constant safety margin of 50%.

$$-0.5F_l \leq F_l \leq 0.5F_l \qquad \forall l,t \qquad (26)$$

Nodal power balances

Kirchhoff's Current Law requires generators and storage units, as well as incoming and outgoing flows over transmission lines to balance inelastic demand at each transmission grid node n and time step t.

$$\sum_{r\in n,c} g_{r,c,t} + \sum_{c} h_{n,c,t}^{\text{discharge}} - \sum_{c} h_{n,c,t}^{\text{charge}} - \sum_{l} K_{nl} f_{l,t} = D_{n,t} \qquad \forall n,t \tag{27}$$

where $D_{n,t}$ is the exogenous inelastic electricity demand and K_{nl} is the incidence matrix of the network. K_{nl} has the value of 1 if the line l starts at the node n, the value of -1 if it ends at the node n, and the value of 0 if it is not connected to the node n at all.

Regional impacts

EXPANSE calculates regional greenhouse gas emissions for each NUTS region r.

$$f_{\text{GHG},r} = \sum_{c,t} w_t g_{r,c,t} i_c^{\text{GHG}} \tag{28}$$

EXPANSE calculates regional particulate matter emissions for each NUTS region r.

$$f_{\text{PM10},r} = \sum_{c,t} w_t g_{r,c,t} i_c^{\text{PM10}} \tag{29}$$

EXPANSE calculates regional jobs associated to generation capacities for each NUTS region r. EXPANSE calculates regional jobs associated to storage capacities for each region r by first calculating jobs at the connected grid node n. These jobs are then evenly divided by the total number m_n of regions that are connected to this grid node. EXPANSE calculates regional jobs associated to transmission lines l in a similar way, but first allocates half of the transmission jobs at each line l to each of the two connected nodes n.

$$f_{\text{Jobs},r} = \sum_{c} G_{r,c} i_c^{\text{Jobs}} + \frac{1}{m_n} \sum_{c} H_{n\in r,c} i_c^{\text{Jobs}} + \frac{1}{2m_n} \sum_{l\in r} F_l L_l i_c^{\text{Jobs}} \tag{30}$$

EXPANSE calculates regional land use for each NUTS region r.

$$f_{\text{LandUse},r} = \sum_{c} G_{r,c} i_c^{\text{LandUse}} + \frac{1}{m_n} \sum_{c} H_{n\in r,c} i_c^{\text{LandUse}} \tag{31}$$

Note on software implementation of EXPANSE

EXPANSE is written with Python 3.7 and employs the pyomo[89] software package (version 6.1.2) to construct the optimization problem. The optimization uses the Gurobi[90] solver.

1.5. Example calculation of regional benefits and vulnerabilities

Calculating average regional benefit regarding employment gains

Here, we present an example for calculating scenario averages of regional benefit for each region r and impact type k by applying Equation (1) of the main manuscript:

$$B_r^k = \frac{1}{N}\sum_t^T \sum_n^N E_{r,n,t}^k$$

In the following example, we calculate average regional benefit B regarding employment increases in the capital region of Brussels (NUTS-2 region code: BE10) for all MGA scenarios and the minimum cost scenario (N = 249) and all technologies of electricity generation, storage, and transmission (T = 21). We assume that in 100 of these 249 scenarios, total employment increases E are equal to 1 job per capita and zero in all other scenarios. With these assumptions, the above equation becomes:

$$B_{\mathrm{BE10}}^{\mathrm{Jobs}} = \frac{1}{249}(100 \cdot 1 + 149 \cdot 0) = 0.401$$

With these assumptions, the average regional benefit regarding employment in the capital region of Brussels is equal to 0.401 jobs per capita.

Calculating average regional vulnerability regarding employment losses

Here, we present an example for calculating scenario averages of regional vulnerability for each region r and impact type k by applying Equation (2) of the main manuscript:

$$V_r^k = \frac{1}{N}\sum_t^T \sum_n^N E_{r,n,t}^k \sum_i^I w_i^k S_{r,i}^k \left[1 - \sum_j^J w_j^k A_{r,j}^k\right]$$

In the following example, we calculate average regional vulnerability V regarding employment losses in the capital region of Brussels. We assume that the normalized adverse impact regarding employment losses is equal to 1 in 100 scenarios and otherwise zero in all other 149 scenarios. We further assume equal weights ($w = 1/3$) for all sensitivity (I = 3) and adaptive capacity (J = 3) indicators. In this example, we assume that all three sensitivity indicators S in the capital region of Brussels are equal to 0.3 and that all three adaptive capacity indicators A are equal to 0.9. With these assumptions, the above equation becomes:

$$V_{\mathrm{BE10}}^{\mathrm{Jobs}} = \frac{1}{249}(100 \cdot 1 + 149 \cdot 0)\left(\frac{1}{3} \cdot 3 \cdot 0.3\right)\left(1 - \frac{1}{3} \cdot 3 \cdot 0.9\right) = 0.012$$

We then normalize this resulting vulnerability to normalized values between 0 (low) and 1 (high) with min-max normalization[54]. If we assume that regional vulnerabilities range between 0.010 and 0.5 across all regions, the normalized vulnerability of the capital region of Brussels becomes:

$$V_{\mathrm{BE10}}^{\mathrm{Jobs}\prime} = \frac{0.012 - 0.010}{0.5 - 0.010} = 0.005$$

With these assumptions, the normalized average regional vulnerability index regarding employment losses in the capital region of Brussels is equal to 0.005.

Composite index of regional vulnerability across all impact types

Here, we present an example for calculating the composite vulnerability index for the capital region of Brussels. We assume that this region has a normalized average regional vulnerability index of $\mathrm{V} = 0.005$ regarding divestment, employment losses, and electricity price increases, a normalized average regional vulnerability index of $\mathrm{V} = 0.2$ for increases in greenhouse gas and particulate matter emissions, and a normalized average regional vulnerability index of $\mathrm{V} = 1.0$ for increased land use. We add all of these six vulnerability indices by applying the weighted-sum method of multi-criteria analysis[91] with equal weights of 1/6.

$$V_{\mathrm{BE10}} = \frac{1}{6}(3 \cdot 0.005 + 2 \cdot 0.2 + 1.0) = 0.235$$

We then calculate the composite vulnerability indices by normalizing the resulting sum to values between 0 (low) and 1 (high) with min-max normalization[54]. For vulnerability indices ranging between 0.1 and 0.8 across regions, the composite vulnerability index becomes:

$$V_{\mathrm{BE10}}{}' = \frac{0.235 - 0.1}{0.8 - 0.1} = 0.192$$

With these assumptions, the capital region of Brussels has a composite vulnerability index of 0.192.

2. Supplementary Figures

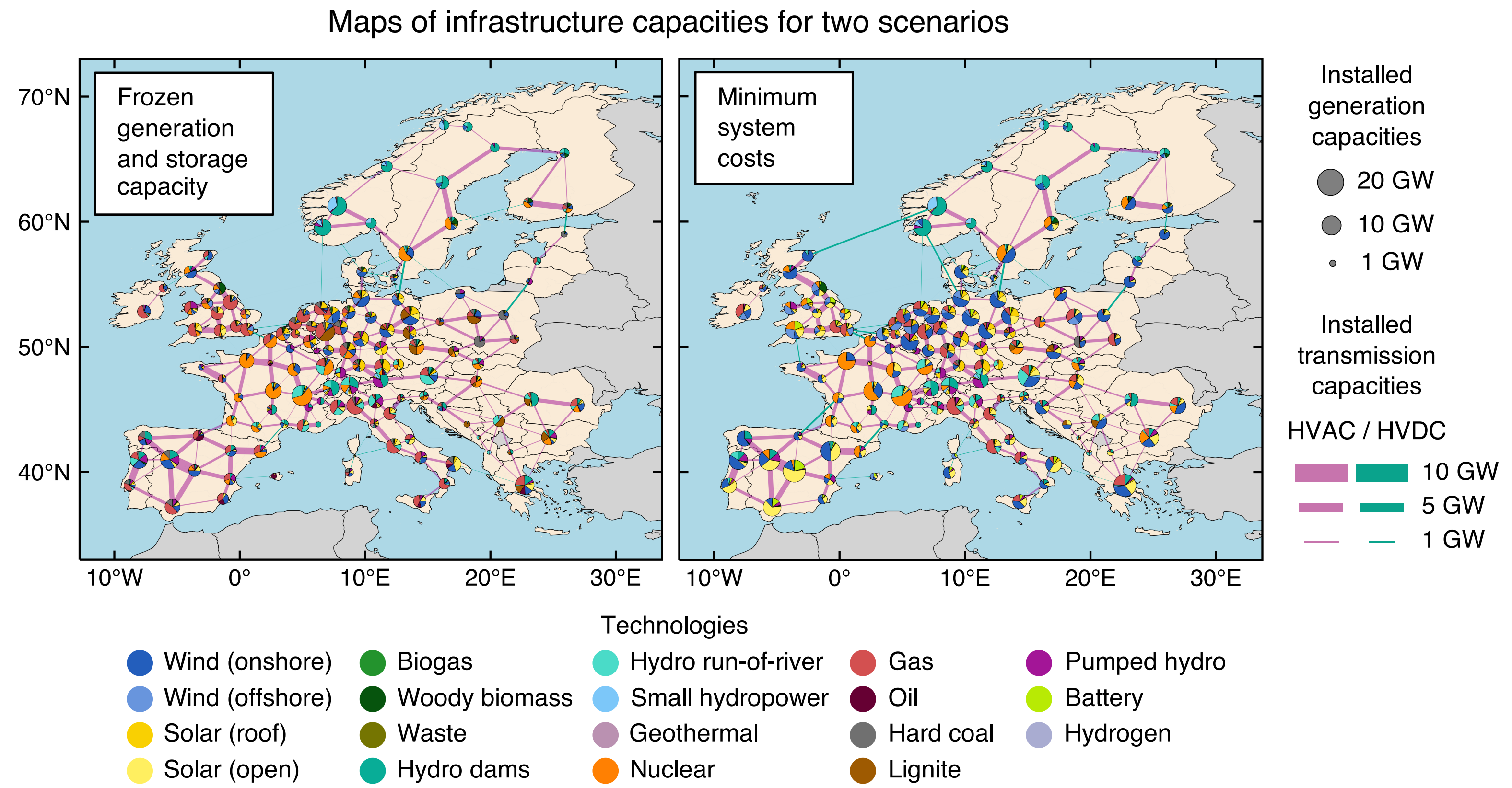

Supplementary Fig. 1: Detailed maps of electricity system infrastructure for two scenarios in 2035. Maps show electricity generation, storage, and transmission capacities for the scenario of frozen generation and storage capacity and the scenario of minimum system costs. The frozen scenario assumes that generation and storage capacities of 2035 are as in 2018, but that transmission capacities can increase to accommodate the higher electricity demand of 2035. The scenario of minimum system costs is found through optimization. For visualization purposes, installed capacities are shown at grid node-level instead of NUTS-2 level[55]. Software to reproduce this figure can be found on Zenodo[92]. HVDC high voltage direct current transmission line; HVAC high voltage alternating current transmission line. Background maps: Made with Natural Earth. Source data are provided with this paper.

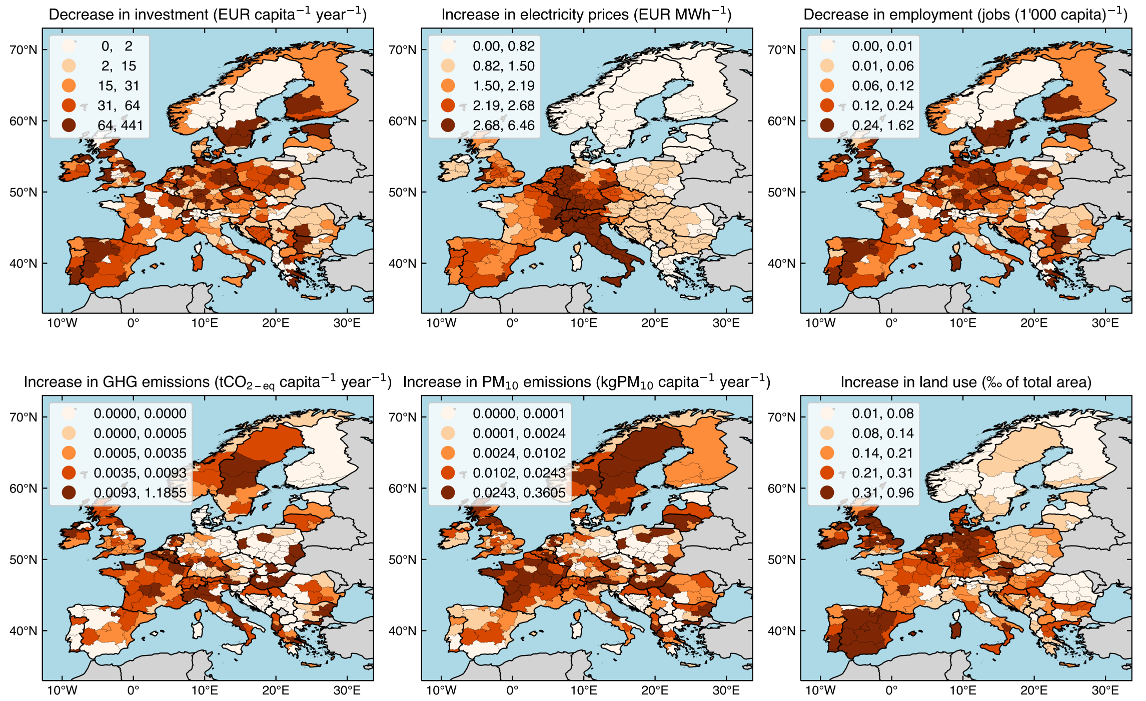

Supplementary Fig. 2: Maps of average regional adverse impacts for all scenarios in 2035. The maps show the average regional adverse impacts across all 248 MGA scenarios and the minimum cost scenario, which have adverse impacts, as compared to the scenario of frozen generation and storage capacity. The color legend uses a quantile classification scheme so that each category has an equal number of regions. Software to reproduce this figure can be found on Zenodo[92]. GHG greenhouse gas emissions; PM particulate matter. Background maps: Made with Natural Earth. Source data are provided with this paper.

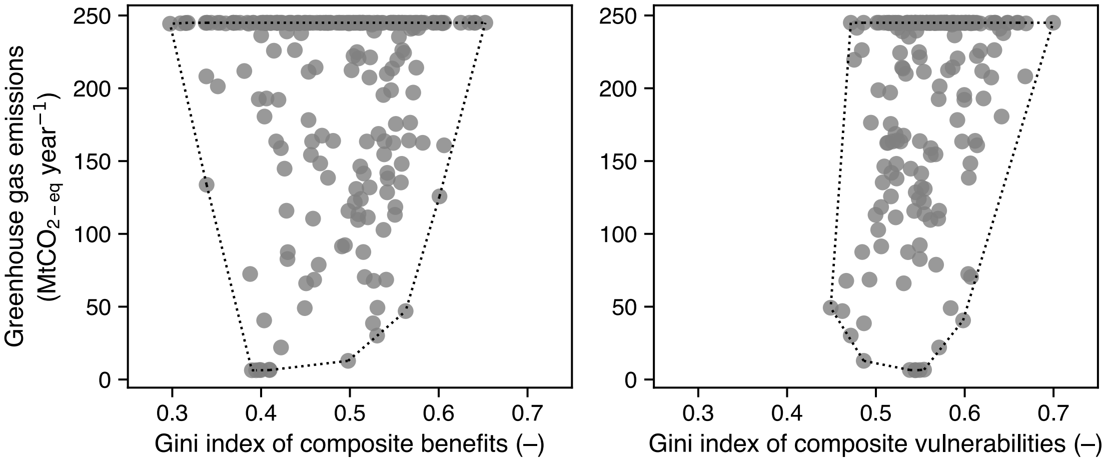

Supplementary Fig. 3: Gini indices of composite regional benefit and vulnerability. Scatter points depict total greenhouse gas emissions and regional inequality of composite regional benefit and vulnerability of all scenarios. Regional inequality is measured with the Gini index[93], where a Gini index of 0 refers to perfect equality and a Gini index of 1 refers to perfect inequality. Dotted lines depict the convex hull[94] of all scatter points. Software to reproduce this figure can be found on Zenodo[92]. Source data are provided with this paper.

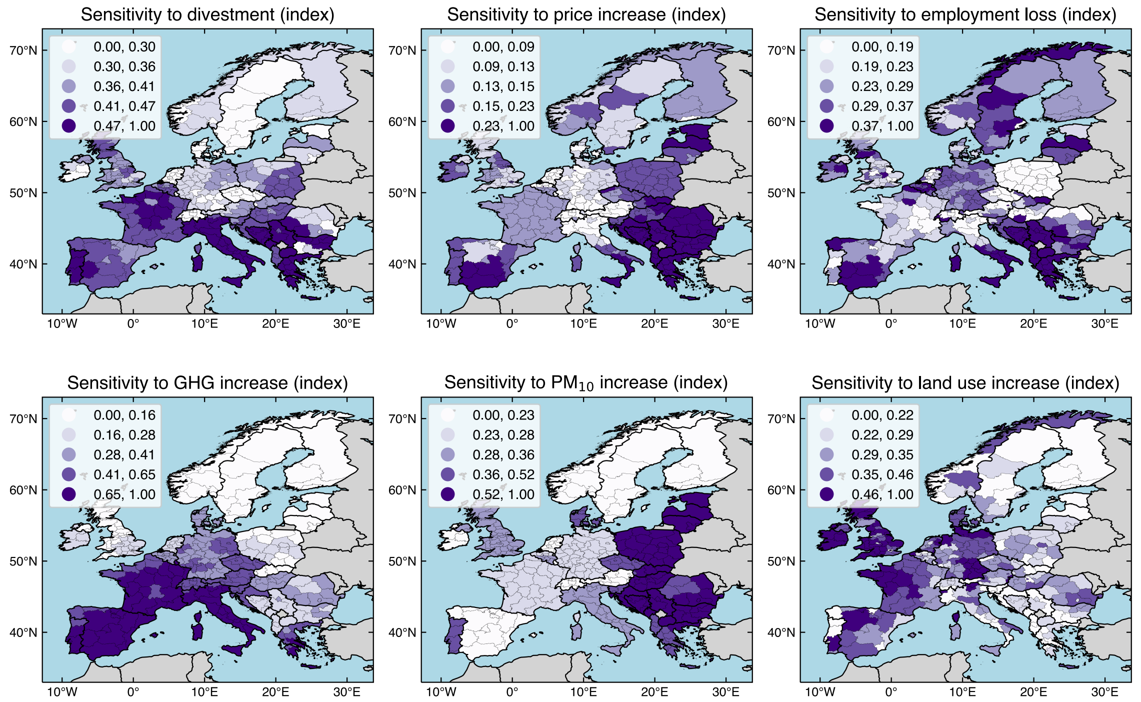

Supplementary Fig. 4: Maps of regional sensitivity to adverse impacts. Sensitivity indexes are calculated with regional statistics data (detailed in Experimental Procedures of the main manuscript) and are scaled to values between 0 (low) and 1 (high). The color legend uses a quantile classification scheme so that each category has an equal number of regions. Software to reproduce this figure can be found on Zenodo[92]. GHG greenhouse gas emissions; PM particulate matter. Background maps: Made with Natural Earth. Source data are provided with this paper.

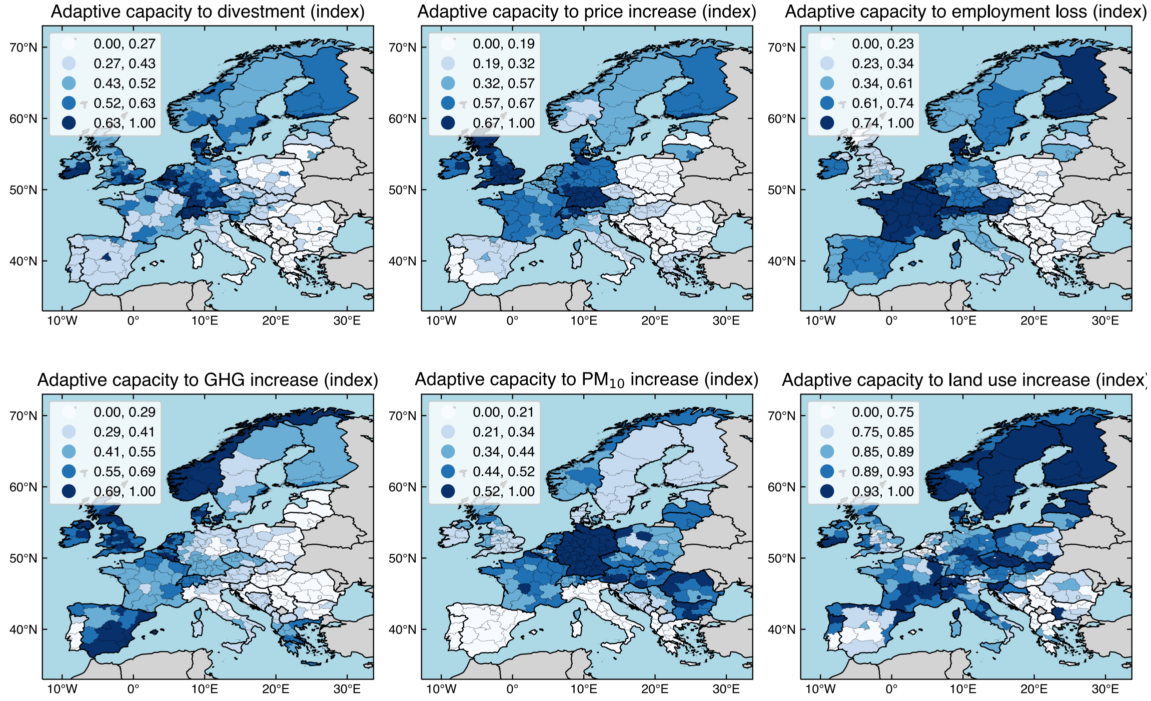

Supplementary Fig. 5: Maps of regional adaptive capacity to adverse impacts. Adaptive capacity indexes are calculated with regional statistics data (detailed in Experimental Procedures of the main manuscript) and are scaled to values between 0 (low) and 1 (high). The color legend uses a quantile classification scheme so that each category has an equal number of regions. Software to reproduce this figure can be found on Zenodo[92]. GHG greenhouse gas emissions; PM particulate matter. Background maps: Made with Natural Earth. Source data are provided with this paper.

Supplementary References

1. Bauer, C. *et al. Potentials, costs and environmental assessment of electricity generation technologies*. (Swiss Federal Office of Energy, Berne, 2017).
2. Rutovitz, J., Dominish, E. & Downes, J. *Calculating global energy sector jobs: 2015 methodology*. (University of Technology Sydney, 2015).
3. Wernet, G. *et al.* The ecoinvent database version 3 (part I): overview and methodology. *Int J Life Cycle Assess* **21**, 1218–1230 (2016).
4. Joint Research Center. *ETRI 2014 - Energy Technology Reference Indicator projections for 2010-2050*. (2014).
5. Luderer, G. *et al.* Environmental co-benefits and adverse side-effects of alternative power sector decarbonization strategies. *Nat Commun* **10**, 5229 (2019).
6. Energy Information Adminstration. Carbon Dioxide Emissions Coefficients. https://www.eia.gov/environment/emissions/co2_vol_mass.php (2021).
7. Jacobson, M. Z. *et al.* Impacts of Green New Deal Energy Plans on Grid Stability, Costs, Jobs, Health, and Climate in 143 Countries. *One Earth* **1**, 449–463 (2019).
8. Aggreko. Y.Cube Energy storage - Product Guide. https://www.aggreko.com/-/media/Aggreko/Files/PDF/Energy-Storage/YCubeProductGuideFinal.pdf (2020).
9. IRENA. *Green Hydrogen Cost Reduction: Scaling up Electrolysers to Meet the 1.5^{0}C Climate Goal*. (International Renewable Energy Agency, Abu Dhabi, 2020).
10. Eurostat. Data from 'Annual detailed enterprise statistics on electricity, gas and water supply (NACE Rev. 1.1, E)'. https://ec.europa.eu/eurostat/databrowser/view/sbs_na_2a_el/default/table (2008).
11. Open Power System Data. Data from 'Data Package Conventional Power Plants'. https://doi.org/10.25832/conventional_power_plants/2020-10-01 (2020).
12. Open Power System Data. Data from 'Data Package Renewable Power Plants'. https://doi.org/10.25832/renewable_power_plants/2020-08-25 (2020).
13. Felice, M. De & Kavvadias, K. Data from 'energy-modelling-toolkit/hydro-power-database: JRC Hydro-power database - release 05'. Zenodo. https://doi.org/10.5281/zenodo.3862722 (2020).
14. International Energy Agency. *Annual data on electricity and heat supply and consumption for OECD and non-OECD countries*. (International Energy Agency, Paris, 2019).
15. Hörsch, J., Hofmann, F., Schlachtberger, D. & Brown, T. PyPSA-Eur: An open optimisation model of the European transmission system. *Energy Strategy Reviews* **22**, 207–215 (2018).
16. Dunnett, S., Sorichetta, A., Taylor, G. & Eigenbrod, F. Harmonised global datasets of wind and solar farm locations and power. *Sci Data* **7**, 130 (2020).
17. Gotzens, F., Heinrichs, H., Hörsch, J. & Hofmann, F. Performing energy modelling exercises in a transparent way - The issue of data quality in power plant databases. *Energy Strategy Reviews* **23**, 1–12 (2019).
18. IG Windkraft. Data from 'Windrad-Landkarte'. https://igwindkraft.at/?mdoc_id=1016663 (2021).
19. Biermayr, P. *et al. Innovative Energietechnologien in Österreich - Marktentwicklung 2018*. (Bundesministerium für Verkehr, Innovation und Technologie, Vienna, 2019).

20. Swiss Federal Office of Energy. Data from 'Feed-in remuneration list of Switzerland'. https://www.bfe.admin.ch/bfe/en/home/promotion/renewable-energy/feed-in-remuneration-at-cost.html (2021).
21. Department of Energy and Climate Change. Data from 'The UK Renewable Energy Planning Database (REPD): March 2020'. https://www.gov.uk/government/publications/renewable-energy-planning-database-monthly-extract (2020).
22. Österreichischer Biomasse-Verband. *Bioenergie Atlas Österreich*. (2019).
23. Elia Group. Data from 'Generating facilities of Belgium'. https://www.elia.be/en/grid-data/power-generation/generating-facilities (2021).
24. The Energy Authority. Data from 'Register of Finnish power plants'. https://energiavirasto.fi/toimitusvarmuus (2021).
25. Ministry of Ecological Transition. Data from 'Renewable electricity by department and by municipality of France'. https://www.statistiques.developpement-durable.gouv.fr/donnees-locales-relatives-aux-installations-de-production-delectricite-renouvelable-beneficiant-0 (2019).
26. Terna. Data from 'List of powerplants in Italy'. http://download.terna.it/terna/0000/0216/18.XLSX (2021).
27. Ministry for Ecological Transition. Data from 'Register of electric power producers of Spain'. https://energia.serviciosmin.gob.es/Electra (2021).
28. Swiss Federal Office of Energy. Electricity production plants. https://www.bfe.admin.ch/bfe/en/home/supply/statistics-and-geodata/geoinformation/geodata/production-plants/electricity-production-plants.html (2020).
29. Tröndle, T., Pfenninger, S. & Lilliestam, J. Home-made or imported: On the possibility for renewable electricity autarky on all scales in Europe. *Energy Strategy Reviews* **26**, 100388 (2019).
30. Ruiz, P. *et al.* ENSPRESO - an open, EU-28 wide, transparent and coherent database of wind, solar and biomass energy potentials. *Energy Strategy Reviews* **26**, 100379 (2019).
31. Limberger, J. *et al.* Assessing the prospective resource base for enhanced geothermal systems in Europe. *Geothermal Energy Science* **2**, 55–71 (2014).
32. European Commission. *Technical Note - Results of the EUCO3232.5 scenario on Member States*. (European Commission, Brussels, 2019).
33. ENTSO-E. *Ten-Year Network Development Plan 2020 – Main Report*. (2020).
34. Tröndle, T., Lilliestam, J., Marelli, S. & Pfenninger, S. Trade-Offs between Geographic Scale, Cost, and Infrastructure Requirements for Fully Renewable Electricity in Europe. *Joule* **4**, 1929–1948 (2020).
35. Pfenninger, S. & Staffell, I. Long-term patterns of European PV output using 30 years of validated hourly reanalysis and satellite data. *Energy* **114**, 1251–1265 (2016).
36. Dee, D. P. *et al.* The ERA-Interim reanalysis: configuration and performance of the data assimilation system. *Quarterly Journal of the Royal Meteorological Society* **137**, 553–597 (2011).

37. Lehner, B. & Grill, G. Global river hydrography and network routing: baseline data and new approaches to study the world's large river systems. *Hydrol Process* **27**, 2171–2186 (2013).
38. IRENA. *Renewable capacity statistics 2018*. (2018).
39. European Commission. National Energy and Climate Plans. https://ec.europa.eu/energy/topics/energy-strategy/national-energy-climate-plans_en (2020).
40. Geth, F., Brijs, T., Kathan, J., Driesen, J. & Belmans, R. An overview of large-scale stationary electricity storage plants in Europe: Current status and new developments. *Renewable and Sustainable Energy Reviews* **52**, 1212–1227 (2015).
41. Eurostat. Data from 'Supply, transformation and consumption of electricity'. https://ec.europa.eu/eurostat/databrowser/view/nrg_cb_e/default/table (2020).
42. Swiss Federal Office of Energy. *Schweizerische Elektrizitätsstatistik 2020*. (Swiss Federal Office of Energy (SFOE), Bern, 2020).
43. IEA. *World Energy Outlook 2019*. (International Energy Agency (IEA), Paris, 2019).
44. Danish Energy Agency. *Technology Data - Generation of electricity and district heating (version 9)*. (2020).
45. European Commission. *ASSET Study on Technology pathways in decarbonisation scenarios*. (2018).
46. Fraunhofer Institute for Solar Energy Systems. *Stromgestehungskosten Erneuerbare Energien*. (2018).
47. Schröder, A., Kunz, F., Meiss, J., Mendelevitch, R. & von Hirschhausen, C. *Current and Prospective Costs of Electricity Generation until 2050*. (German Institute for Economic Research, Berlin, 2013).
48. Collins, S., Deane, P., Ó Gallachóir, B., Pfenninger, S. & Staffell, I. Impacts of Inter-annual Wind and Solar Variations on the European Power System. *Joule* **2**, 2076–2090 (2018).
49. Schmidt, O. *et al.* Projecting the Future Levelized Cost of Electricity Storage Technologies. *Joule* **3**, 81–100 (2019).
50. Hagspiel, S. *et al.* Cost-optimal power system extension under flow-based market coupling. *Energy* **66**, 654–666 (2014).
51. European Commission. *The European Green Deal. COM(2019) 640 final*. (2019).
52. European Commission. *Stepping up Europe's 2030 climate ambition - Investing in a climate-neutral future for the benefit of our people*. (2020).
53. Pietzcker, R. C., Osorio, S. & Rodrigues, R. Tightening EU ETS targets in line with the European Green Deal: Impacts on the decarbonization of the EU power sector. *Appl Energy* **293**, 116914 (2021).
54. Suarez-Alvarez, M. M., Pham, D.-T., Prostov, M. Y. & Prostov, Y. I. Statistical approach to normalization of feature vectors and clustering of mixed datasets. *Proceedings of the Royal Society A: Mathematical, Physical and Engineering Sciences* **468**, 2630–2651 (2012).
55. European Parliament. *Regulation (EC) No 1059/2003 of the European Parliament and of the Council of 26 May 2003 on the Establishment of a Common Classification of Territorial Units for Statistics (NUTS)*. (European Parliament, Brussels, 2019).

56. Eurostat. *Labour costs survey 2008, 2012 and 2016 - NACE Rev. 2 activity*. (Eurostat, Luxembourg, 2016).
57. Eurostat. *Government deficit/surplus, debt and associated data*. (Eurostat, Luxembourg, 2019).
58. Eurostat. *Gross domestic product (GDP) at current market prices by NUTS 2 regions*. (Eurostat, Luxembourg, 2018).
59. Eurostat. *Arrears on utility bills - EU-SILC survey*. (Eurostat, Luxembourg, 2019).
60. Eurostat. *Structure of consumption expenditure by COICOP consumption purpose*. (Eurostat, Luxembourg, 2015).
61. Eurostat. *People at risk of poverty or social exclusion by NUTS regions*. (Eurostat, Luxembourg, 2019).
62. Eurostat. *Long-term unemployment (12 months and more) by sex, age, educational attainment level and NUTS 2 regions (%)*. (Eurostat, Luxembourg, 2019).
63. Eurostat. *SBS data by NUTS 2 regions and NACE Rev. 2 (from 2008 onwards)*. (Eurostat, Luxembourg, 2018).
64. Eurostat. *People living in households with very low work intensity by NUTS 2 regions (population aged 0 to 59 years)*. (Eurostat, Luxembourg, 2019).
65. European Environment Agency. *Unequal exposure and unequal impacts: social vulnerability to air pollution, noise and extreme temperatures in Europe*. (Publications Office of the European Union, Luxembourg, 2018).
66. European Environment Agency. *Climate change adaptation and disaster risk reduction in Europe: enhancing coherence of the knowledge base, policies and practices*. (Publications Office of the European Union, Luxembourg, 2017).
67. Eurostat. *Self-reported unmet needs for medical examination by main reason declared and NUTS 2 regions*. (Eurostat, Luxembourg, 2019).
68. Eurostat. *Self-perceived health by sex, age and degree of urbanisation*. (Eurostat, Luxembourg, 2019).
69. Eurostat. *Population density by NUTS 2 region*. (Eurostat, Luxembourg, 2018).
70. Eurostat. *Arrivals at tourist accommodation establishments by NUTS 2 regions*. (Eurostat, Luxembourg, 2019).
71. Eurostat. *Key variables: area, livestock (LSU), labour force and standard output (SO) by economic size of farm (SO in Euro), legal status of holding and NUTS 2 regions*. (Eurostat, Luxembourg, 2013).
72. Eurostat. *Employment in high-tech sectors by NUTS 2 regions*. (Eurostat, Luxembourg, 2019).
73. Eurostat. *Road, rail and navigable inland waterways networks by NUTS 2 regions*. (Eurostat, Luxembourg, 2019).
74. Charron, N., Lapuente, V. & Annoni, P. Measuring quality of government in EU regions across space and time. *Papers in Regional Science* **98**, 1925–1953 (2019).
75. Eurostat. *Expenditure: main results*. (Eurostat, Luxembourg, 2018).
76. Eurostat. *Income of households by NUTS 2 regions*. (Eurostat, Luxembourg, 2017).
77. European Commission. *Comprehensive study of building energy renovation activities and the uptake of nearly zero-energy buildings in the EU*. (Publications Office of the European Union, Luxembourg, 2019).

78. European Commission. *LMP expenditure by type of action - summary tables*. (European Commission, Brussels, 2018).
79. Eurostat. *Employment rates by sex, age, educational attainment level, citizenship and NUTS 2 regions*. (Eurostat, Luxembourg, 2019).
80. Eurostat. *Population on 1 January by age group, sex and NUTS 2 region*. (Eurostat, Luxembourg, 2019).
81. Eurostat. *EU-SILC Module 2007 On Housing Conditions*. (Eurostat, Luxembourg, 2009).
82. Eurostat. *Hospital beds by NUTS 2 regions*. (Eurostat, Luxembourg, 2018).
83. Eurostat. *Life expectancy by age, sex and NUTS 2 region*. (Eurostat, Luxembourg, 2018).
84. European Environment Agency. *Corine Land Cover (CLC) 2018*. (European Environment Agency, Copenhagen, 2020).
85. Eurostat. *Gross value added at basic prices by NUTS 3 regions*. (Eurostat, Luxembourg, 2018).
86. Li, F. G. N. & Trutnevyte, E. Investment appraisal of cost-optimal and near-optimal pathways for the UK electricity sector transition to 2050. *Appl Energy* **189**, 89–109 (2017).
87. Berkelaar, M., Eikland, K. & Notebaert, P. Absolute values. *lp_solve* http://lpsolve.sourceforge.net/5.5/absolute.htm (2004).
88. Trutnevyte, E. Does cost optimization approximate the real-world energy transition? *Energy* **106**, 182–193 (2016).
89. Hart, W. E., Watson, J.-P. & Woodruff, D. L. Pyomo: modeling and solving mathematical programs in Python. *Math Program Comput* **3**, 219–260 (2011).
90. Gurobi optimization. Gurobi Optimizer Reference Manual. Preprint at (2021).
91. Belton, V. & Stewart, T. J. *Multiple Criteria Decision Analysis: An Integrated Approach*. (Kluwer Academic Publishers Group, Dordrecht, 2002).
92. Sasse, J.-P. & Trutnevyte, E. Software related to Sasse et al. (2023) 'A low-carbon electricity sector in Europe risks sustaining regional inequalities in benefits and vulnerabilities'. *Zenodo* (2023) doi:10.5281/ZENODO.7777215.
93. Gini, C. *Variabilità e mutabilità*. (Libreria Eredi Virgilio Veschi, Rome, 1912).
94. Barber, C. B., Dobkin, D. P. & Huhdanpaa, H. The Quickhull Algorithm for Convex Hulls. *ACM Transactions on Mathematical Software* **22**, 469–483 (1996).
