# Supplementary material for: A low-carbon electricity sector in Europe risks sustaining regional inequalities in benefits and vulnerabilities
Source: Nat Commun. 2023 Apr 18;14:2205. doi: 10.1038/s41467-023-37946-3 (PMC10111333; doi:10.1038/s41467-023-37946-3)
Supplement: Supplementary file 2 — Reporting Summary [file 41467_2023_37946_MOESM2_ESM.pdf]

Corresponding author(s): Jan-Philipp Sasse

Last updated by author(s): Mar 29, 2023

Reporting Summary

Nature Portfolio wishes to improve the reproducibility of the work that we publish. This form provides structure for consistency and transparency in reporting. For further information on Nature Portfolio policies, see our Editorial Policies and the Editorial Policy Checklist.

Statistics

For all statistical analyses, confirm that the following items are present in the figure legend, table legend, main text, or Methods section.

n/a	Confirmed	
☒	☐	The exact sample size (*n*) for each experimental group/condition, given as a discrete number and unit of measurement
☒	☐	A statement on whether measurements were taken from distinct samples or whether the same sample was measured repeatedly
☒	☐	The statistical test(s) used AND whether they are one- or two-sided<br>*Only common tests should be described solely by name; describe more complex techniques in the Methods section.*
☒	☐	A description of all covariates tested
☒	☐	A description of any assumptions or corrections, such as tests of normality and adjustment for multiple comparisons
☐	☒	A full description of the statistical parameters including central tendency (e.g. means) or other basic estimates (e.g. regression coefficient) AND variation (e.g. standard deviation) or associated estimates of uncertainty (e.g. confidence intervals)
☒	☐	For null hypothesis testing, the test statistic (e.g. *F*, *t*, *r*) with confidence intervals, effect sizes, degrees of freedom and *P* value noted<br>*Give P values as exact values whenever suitable.*
☒	☐	For Bayesian analysis, information on the choice of priors and Markov chain Monte Carlo settings
☒	☐	For hierarchical and complex designs, identification of the appropriate level for tests and full reporting of outcomes
☒	☐	Estimates of effect sizes (e.g. Cohen's *d*, Pearson's *r*), indicating how they were calculated

Our web collection on statistics for biologists contains articles on many of the points above.

Software and code

Policy information about availability of computer code

Data collection: No software was used for data collection.

Data analysis: The data analysis code to process and plot all result data is publicly available on Zenodo: https://doi.org/10.5281/zenodo.7777215.

We applied the EXPANSE model, which has been extensively described in peer-reviewed literature.
Sasse, JP. & Trutnevyte, E. Distributional trade-offs between regionally equitable and cost-efficient allocation of renewable electricity generation. Appl. Energy 254, (2019).
Sasse, JP., Trutnevyte, E. Regional impacts of electricity system transition in Central Europe until 2035. Nat Commun 11, 4972 (2020).

All equations of the EXPANSE model are presented in Supplementary Methods. EXPANSE applies the Pyomo optimization toolbox. Pyomo (Version 6.1.2): https://github.com/Pyomo/pyomo. The optimization uses the Gurobi solver. Gurobi (Version 9.5.0): https://www.gurobi.com.

We performed all spatial data analysis with the Geopandas package, and data processing with the Pandas package.
Geopandas (Version 0.10.2): https://github.com/geopandas/geopandas
Pandas (Version 1.3.4): https://github.com/pandas-dev/pandas

Background maps were added for visualization purposes by applying the Cartopy package.
Cartopy (Version 0.21.1): https://github.com/SciTools/cartopy

For manuscripts utilizing custom algorithms or software that are central to the research but not yet described in published literature, software must be made available to editors and reviewers. We strongly encourage code deposition in a community repository (e.g. GitHub). See the Nature Portfolio guidelines for submitting code & software for further information.

Data

Policy information about availability of data

All manuscripts must include a data availability statement. This statement should provide the following information, where applicable:

- Accession codes, unique identifiers, or web links for publicly available datasets
- A description of any restrictions on data availability
- For clinical datasets or third party data, please ensure that the statement adheres to our policy

All input data are either provided in the Supplementary Information or come from openly accessible public repositories that are cited. All result data of this study are provided in Supplementary Data and are publicly available on Zenodo: https://doi.org/10.5281/zenodo.7777215.

Data on existing conventional power plants are publicly accessible from Open Power System Data: https://doi.org/10.25832/conventional_power_plants/2020-10-01

Data on existing renewable (excluding hydro power) are publicly accessible from Open Power System Data: https://doi.org/10.25832/renewable_power_plants/2020-08-25

Data on existing hydro power plants are publicly accessible from the JRC hydro-power database: https://doi.org/10.5281/zenodo.3862722

Data on existing and potential transmission infrastructure are publicly accessible from the European PyPSA dataset: https://doi.org/10.5281/zenodo.3886532

Data on installed generation capacity potentials of wind and solar PV are publicly accessible from a European modeling study: https://doi.org/10.5281/zenodo.3533038

Data on installed generation capacity potentials of biomass are publicly accessible from the JRC ENSPRESO database: http://data.europa.eu/89h/74ed5a04-7d74-4807-9eab-b94774309d9f

Data on installed generation capacity potentials of geothermal power are publicly accessible from a European modeling study: https://doi.org/10.5194/gtes-2-55-2014

Data on capacity factor time series for solar PV, wind, and hydro power are publicly accessible from a European modeling study: https://doi.org/10.5281/zenodo.3949553

Data on regional statistics to compute indicators of sensitivity and adaptive capacity are publicly accessible on the Eurostat database: https://ec.europa.eu/eurostat/data/database

Human research participants

Policy information about studies involving human research participants and Sex and Gender in Research.

Reporting on sex and gender	N/A
Population characteristics	N/A
Recruitment	N/A
Ethics oversight	N/A

Note that full information on the approval of the study protocol must also be provided in the manuscript.

Field-specific reporting

Please select the one below that is the best fit for your research. If you are not sure, read the appropriate sections before making your selection.

☐ Life sciences ☐ Behavioural & social sciences ☒ Ecological, evolutionary & environmental sciences

For a reference copy of the document with all sections, see nature.com/documents/nr-reporting-summary-flat.pdf

Ecological, evolutionary & environmental sciences study design

All studies must disclose on these points even when the disclosure is negative.

Study description	This is a quantitative study that applies an electricity system model to quantify regional benefits and vulnerabilities of the low-carbon electricity system transition in Europe. We use publicly available data, which are described in the manuscript and supplementary information. We apply a peer-reviewed electricity system model.

Research sample	First, data on existing and potential electricity system infrastructure was collected from several European modeling studies (detailed in "Data" section above and Supplementary Information of this publication). We then ran a total of 250 scenarios of the European electricity system in 2035 by applying the EXPANSE model. These scenarios include one frozen scenario that assumes same generation and storage capacities of 2018 in 2035, a scenario of minimum system costs, and 248 alternative spatial configurations (MGA scenarios) of the electricity system with up 20% higher total system costs as compared to the minimum cost scenario. We choose this number of MGA scenarios to follow other MGA studies with similar spatial and temporal detail. Computing even more MGA scenarios is bounded by the computational tractability of spatially and temporally detailed, continent-scale electricity system models, such as EXPANSE. Each MGA scenario represents an alternative configuration of the electricity system with near-optimal total system costs. These MGA scenarios also represent alternative scenarios with different regional impacts that are within modeling constraints (e.g., operational feasibility of electricity system, maximum limits on greenhouse gas emissions). By applying impact factors (see Supplementary Table 1) on quantities of capacity and operation of electricity system infrastructure, we then computed regional impacts across NUTS2 regions (e.g., employment, land use). By comparing impacts of the frozen scenario with the minimum cost and MGA scenarios, we computed positive impacts (i.e., benefits) and negative impacts (i.e., adverse impacts). We then combined modeled adverse impacts with min-max normalized quantities of region and impact-specific indicators for sensitivity and adaptive capacity, which we derived from regional statistics data (see Eurostat database mentioned in "Data" section above), to quantify region and impact-specific measures of vulnerability.
Sampling strategy	We applied MGA method to compute 248 alternative MGA scenarios where the slack is randomly varied from 0 to 20% above cost-optimal total system costs. We choose this number of MGA scenarios to follow other MGA studies with similar spatial and temporal detail. Computing even more MGA scenarios is bounded by the computational tractability of spatially and temporally detailed, continent-scale electricity system models, such as EXPANSE. Total system costs are defined as the sum of annualized capital and variable costs for generation, storage, and transmission. We select a maximum slack of 20% based on the cost deviations found in a retrospective modeling study and from other forward-looking MGA models. To ensure the spread and diversity of MGA scenarios not only in terms of total costs, but also other impacts analyzed in this study, we adapt previous MGA algorithms to apply computer-generated random objective functions for investment variables (i.e., country and technology-specific generation capacities) and computer-generated random constraints on five continent-wide impact objectives (i.e., minimizing total system costs, greenhouse gas emissions, particulate matter emissions, and land use, and maximizing total employment). For example, the objective of one MGA scenario in terms of investment could be to maximize total offshore wind capacity in Denmark and to minimize total nuclear capacity in Switzerland, while meeting computer-generated random constraints for all five impact objectives. Thus, we extend the MGA approach for a single cost objective (i.e., replacing the cost objective with a cost constraint) to multiple impact objectives (i.e., replacing five impact objectives with five impact constraints).
Data collection	Direct download of datasets from public data repositories (documented in "Data" section above and Supplementary Information).
Timing and spatial scale	All input data was collected in the period between June 2020 and April 2021. The rationale was to use the newest available data for existing and potential electricity system infrastructure, as well as regional statistics data to compute sensitivity and adaptive capacity indicators. All regional benefits and adverse impacts are computed at NUTS-2 spatial level. All sensitivity and adaptive capacity indicators are computed at the NUTS spatial level indicated in Supplementary Tables 6 and 7.
Data exclusions	No data was excluded from the analysis.
Reproducibility	We provide instructions on how data was collected in the Methods and Supplementary Methods sections. All model equations are documented in Supplementary Methods. Software that we used to analyze and plot all results of this study are publicly available on Zenodo: https://doi.org/10.5281/zenodo.7777215.
Randomization	We did not apply randomization because we report results on all 250 scenarios that we model and not on selected scenario groups.
Blinding	We did not apply blinding because we report results on all 250 scenarios that we model and not on selected scenario groups.

Did the study involve field work? ☐ Yes ☒ No

Reporting for specific materials, systems and methods

We require information from authors about some types of materials, experimental systems and methods used in many studies. Here, indicate whether each material, system or method listed is relevant to your study. If you are not sure if a list item applies to your research, read the appropriate section before selecting a response.

Materials & experimental systems

n/a	Involved in the study	
☒	☐	Antibodies
☒	☐	Eukaryotic cell lines
☒	☐	Palaeontology and archaeology
☒	☐	Animals and other organisms
☒	☐	Clinical data
☒	☐	Dual use research of concern

Methods

n/a	Involved in the study	
☒	☐	ChIP-seq
☒	☐	Flow cytometry
☒	☐	MRI-based neuroimaging
